# Microglial debris is cleared by astrocytes via C4b-facilitated phagocytosis and degraded via RUBICON-dependent noncanonical autophagy in mice

**DOI:** 10.1038/s41467-022-33932-3

**Published:** 2022-10-24

**Authors:** Tian Zhou, Yuxin Li, Xiaoyu Li, Fanzhuo Zeng, Yanxia Rao, Yang He, Yafei Wang, Meizhen Liu, Dali Li, Zhen Xu, Xin Zhou, Siling Du, Fugui Niu, Jiyun Peng, Xifan Mei, Sheng-Jian Ji, Yousheng Shu, Wei Lu, Feifan Guo, Tianzhun Wu, Ti-Fei Yuan, Ying Mao, Bo Peng

**Affiliations:** 1 Shenzhen Institute of Advanced Technology, Chinese Academy of Sciences, Shenzhen, Guangdong 518055 China; 2 Department of Neurology, Jinshan Hospital, Institute for Translational Brain Research, State Key Laboratory of Medical Neurobiology, MOE Frontiers Center for Brain Science, Fudan University, Shanghai, 201508 China; 3 Department of Neurosurgery, Huashan Hospital, Fudan University, Shanghai, 200040 China; 4 Department of Orthopedics, The Third Affiliated Hospital of Jinzhou Medical University, Jinzhou, Liaoning 121012 China; 5 Department of Laboratory Animal Science, MOE Frontiers Center for Brain Science, Fudan University, Shanghai, 200032 China; 6 Shanghai Key Laboratory of Psychotic Disorders, Shanghai Mental Health Center, Shanghai Jiao Tong University School of Medicine, Shanghai, 201108 China; 7 Shanghai Frontiers Science Center of Genome Editing and Cell Therapy, Shanghai Key Laboratory of Regulatory Biology and School of Life Sciences, East China Normal University, Shanghai, 200241 China; 8 School of Life Sciences, Department of Biology, Shenzhen Key Laboratory of Gene Regulation and Systems Biology, Brain Research Center, Southern University of Science and Technology, Shenzhen, Guangdong 518055 China; 9 Institute of Life Science, Nanchang University, Nanchang, Jiangxi 330031 China; 10 Co-Innovation Center of Neuroregeneration, Nantong University, Nantong, Jiangsu 226001 China

**Keywords:** Cellular neuroscience, Microglia, Astrocyte

## Abstract

Microglia are important immune cells in the central nervous system (CNS) that undergo turnover throughout the lifespan. If microglial debris is not removed in a timely manner, accumulated debris may influence CNS function. Clearance of microglial debris is crucial for CNS homeostasis. However, underlying mechanisms remain obscure. We here investigate how dead microglia are removed. We find that although microglia can phagocytose microglial debris in vitro, the territory-dependent competition hinders the microglia-to-microglial debris engulfment in vivo. In contrast, microglial debris is mainly phagocytosed by astrocytes in the brain, facilitated by C4b opsonization. The engulfed microglial fragments are then degraded in astrocytes via RUBICON-dependent LC3-associated phagocytosis (LAP), a form of noncanonical autophagy. Interference with C4b-mediated engulfment and subsequent LAP disrupt the removal and degradation of microglial debris, respectively. Together, we elucidate the cellular and molecular mechanisms of microglial debris removal in mice, extending the knowledge on the maintenance of CNS homeostasis.

## Abstract (web summary)

Microglia are professional phagocytes in the CNS. However, which cells scavenge corpses of microglia is largely neglected. Peng and colleagues found that nonprofessional phagocytes (astrocyte) phagocytose the debris of professional phagocytes (microglia).

## Introduction

Microglia are resident immune cells in the central nervous system (CNS) that play vital roles in CNS development, homeostasis and disease^1,2^. As professional phagocytes, microglia engulf cell corpses, excessive cells, invading pathogens and amyloid beta (Aβ)^3–15^. Efficient phagocytosis is essential for maintenance of the homeostasis of the CNS^3–6,9,10,15^.

Different from neurons that are lifelong surviving in the adulthood, yolk sac-derived microglia undergo constant turnover. During this process, old microglia die, and new microglia regenerate via self-renewal^16–21^. The generation of newly formed microglia is coupled with the apoptosis of dysfunctional microglia to maintain the stability of the cell population^22^. In the homeostatic CNS, approximately 30% of microglia turn over each year in both mouse and human brains^20,23^. In other words, approximately one-third of microglia die in the homeostatic brain each year. The speed of microglial turnover in diseased CNS is even faster than that in healthy CNS^23,24^. If the cellular corpses are not removed in a timely manner, the accumulated debris can interfere with CNS function^25,26^. Although microglia are the major cells responsible for the removal of cell debris, microglia per se are unlikely to phagocytose their congeneric corpses. In an extreme condition of rapid microglial depletion, more than 95% of CNS microglia are ablated within a few days. However, we and other research groups have found that rapid microglial depletion does not result in massive microglial debris accumulation or detectable inflammatory responses^27–30^. Furthermore, even in the retina, where all microglia are pharmacologically depleted, no massive accumulation of microglial debris has been observed^31^. The residual microglia are thus unable to rapidly clear debris due to their low number or even absence. This convergent evidence suggests an unknown yet efficient machinery that governs microglial debris removal. Therefore, investigating the removal of microglial debris is important for understanding the maintenance of CNS homeostasis. Nevertheless, the clearance of microglial debris is almost neglected, probably due to the primary scavenger role of microglia per se.

To this end, we studied the cellular and molecular mechanisms underlying microglial debris removal. We first screened the major cell types in the brain as potential candidates, including astrocytes, pericytes, endothelial cells, vascular smooth muscle cells (VSMCs), oligodendrocyte precursor cells (OPCs), oligodendrocytes, neurons, neural stem cells (NSCs), microglia and CNS border-associated macrophages (BAMs). We found that pericytes, endothelial cells, VSMCs, OPCs, oligodendrocytes, neurons, NSCs and BAMs do not engulf microglial debris in vivo. Instead, microglial debris is primarily removed by astrocytes, a nonprofessional phagocyte, with a noncanonical and sophisticated reactive state. The astrocyte phagocytosis rates are positively correlated with the speed of microglial turnover under both physiological and pathological conditions. Although in vitro experiments indicate that microglia are able to engulf their congeneric debris, a territory-dependent competition of astrocytes hinders microglia-to-microglial debris engulfment in vivo. We also demonstrated that the astrocytic engulfment of microglial debris is facilitated through a C4b opsonization mechanism, and the engulfed microglial debris is subsequently degraded via LC3-associated phagocytosis (LAP), a form of noncanonical autophagy. The debris-containing LC3-associated phagosomes (LAPosomes) are then fused with lysosomes to form phagolysosomes for further degradation. The interference of LAP formation disrupts the degradation of engulfed microglial debris and induces accumulation of nondegraded debris in astrocytes, which indicates the essential role in the maintenance of CNS homeostasis.

Collectively, our results uncover the phagocytic role of astrocytes in the scavenging of microglial debris. As nonprofessional phagocytes, astrocytes reportedly engulf neuronal debris^32–34^. In this study, we extended this knowledge to the neglected facet of microglial debris clearance. Our study further identified the underlying mechanisms of astrocytic phagocytosis and intracellular degradation of microglial debris. Efficient removal by astrocytes avoids the accumulation of microglial debris. In conclusion, nonprofessional phagocytes phagocytose the debris of professional phagocytes via noncanonical autophagy for degradation. During this process, astrocytes exhibit a noncanonical reactivation phenotype. This study sheds new light on the maintenance of CNS homeostasis.

## Results

### Microglial debris is primarily scavenged by astrocytes in vivo and in vitro

**Fig. 1:**
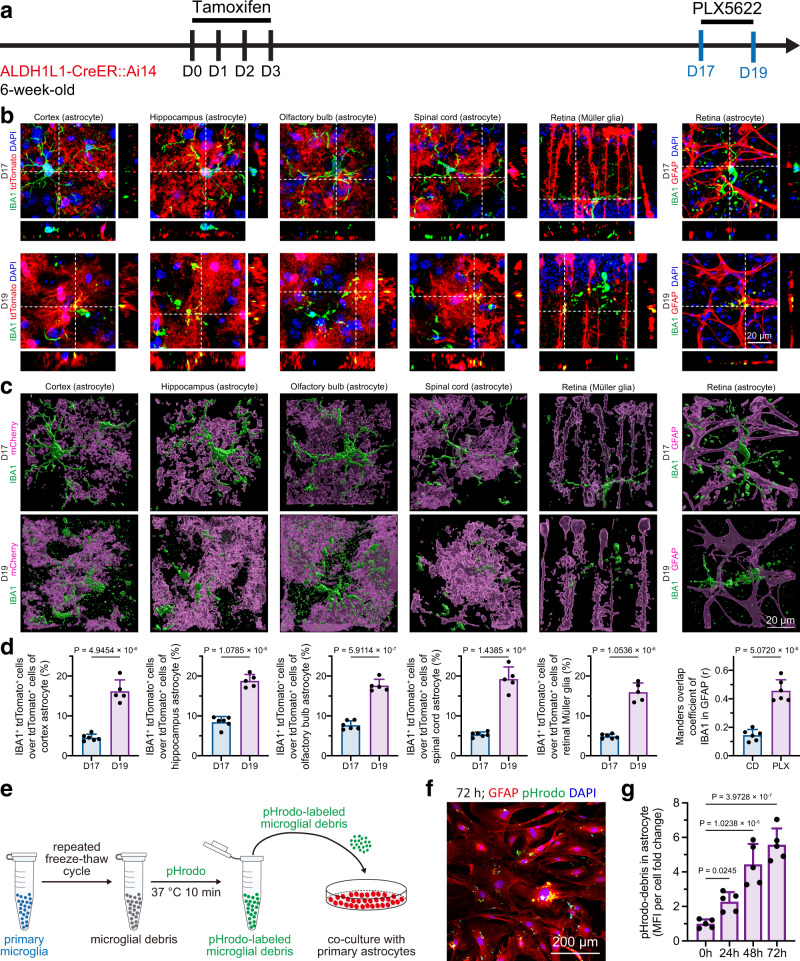
In vivo and in vitro evidence demonstrates that astrocytes (in the brain, spinal cord and retina) and Müller glia (in the retina) are capable of engulfing microglial debris. **a** Scheme of the in vivo astrocytic engulfment examination by ALDH1L1-CreER::Ai14-based astrocyte labeling and microglial depletion. Because ALDL1L1-CreER::Ai14 does not target retinal astrocytes, retinal astrocytes are visualized by GFAP in C57BL/6 J mice treated with CD or PLX5622 for 2 days. **b**, **c** Confocal orthogonal colocalization (**b**) and 3D reconstruction (**c**) show that tdTomato^+^ astrocytes (in the brain, spinal cord and retina) or Müller glia (in the retina) do not engulf IBA1^+^ microglial debris under physiological conditions (D17) whereas they engulf IBA1^+^ microglial debris upon the CSF1R inhibition (D19). **d** Quantification of microglial debris engulfment by astrocytes (in the brain, spinal cord and retina) and Müller glia (in the retina). *N* = 6 mice at D17, 5 mice at D19. Two-tailed independent *t* test. **e** Scheme of the in vitro astrocytic engulfment assay using pHrodo-labeled microglial debris. **f** GFAP^+^ astrocytes engulf pHrodo-labeled microglial debris in FBS-containing culture medium. pHrodo is illustrated by the green pseudocolor for better visualization. **g** Quantification of astrocytic engulfment after exposure to pHrodo-labeled microglial debris for 0, 24, 48, and 72 h. *N* = 5 independent biological replicates for each group. One-way ANOVA with Holm‒Sidak’s multiple comparisons test (post hoc). PLX5622: PLX5622-formulated AIN-76A diet; CD: control AIN-76A diet; PLX: PLX5622; MFI mean fluorescence intensity. Data are presented as mean ± SD. The source data are provided as a Source Data file.

We first questioned which CNS cells are able to engulf microglial debris. In the homeostatic brain, microglial turnover is relatively slow, approximately 0.1% per day^20,23^. The low turnover rate to some extent hinders researchers from studying the clearance of microglial debris. To overcome this obstacle, we utilized PLX5622, a colony-stimulating factor 1 receptor (CSF1R) inhibitor, to accelerate microglial cell death^27,31,35,36^. We administered a PLX5622-formulated AIN-76A diet (1.2 g PLX5622 per kilogram of diet; referred to hereafter as PLX5622) or an AIN-76A control diet (CD) for 2 days to CX3CR1^+/GFP^ mice, in which almost all microglia expressed GFP^37^ (Supplementary Fig. 1a). The dying microglia became collapsed and fragmented upon PLX5622 administration (Supplementary Fig. 2). Using this accelerated cell death model to facilitate the investigation of microglial debris removal, we detected evident GFP^+^ microglial debris incorporated with the astrocyte marker GFAP in the PLX5622-treated brain (Supplementary Fig. 1b). GFAP is an intermediate filament protein that does not illustrate the whole morphology of astrocytes. To better visualize the morphology and rigidly observe the colocalization, we crossed ALDH1L1-CreER mice, a brain and spinal cord astrocyte-specific mouse line^38^, with Ai14 mice to obtain the ALDH1L1-CreER::Ai14 line. Tamoxifen was applied to induce tdTomato expression in brain and spinal cord astrocytes. Two weeks after the final dose, PLX5622 was administered for two days to kill CNS microglia (Fig. 1a). Consistent with our GFAP observations, a high amount of IBA1^+^ microglial debris was colocalized with tdTomato^+^ astrocytes in the brain (Fig. 1b–d and Supplementary Fig. 3). In addition, we intravenously introduced the blood‒brain barrier (BBB)-permeable AAV-PHP.eB^39^ *Gfap*-mCherry, which targets brain astrocytes and drives the fluorescent reporter to fully trace astrocytic morphology, to C57BL/6 J mice (Supplementary Fig. 4a). Thirty days after transduction, the astrocyte-labeled mice were administered PLX5622 for 2 or 4 days to kill brain microglia (Supplementary Fig. 4a). Similar to the observations from ALDL1L1-CreER::Ai14 mice, GFP^+^ microglial debris was colocalized with mCherry^+^ astrocytes in the brain (Supplementary Fig. 4b-c and Supplementary Fig. 5).

The CNS includes the brain, spinal cord and retina. We thus asked whether astrocytes in the spinal cord and retina are also able to engulf microglial debris. We found that spinal cord astrocytes exhibited a similar capability of engulfing microglial debris (Fig. 1b–d and Supplementary Fig. 3). The retina contains two counterparts of brain astrocytes: Müller glia and retinal astrocytes^40^. Müller glia vertically cross the whole retinal layers with their cell bodies localized in the inner nuclear layer (INL), whereas retinal astrocytes horizontally cross the retinal ganglion cell layer (GCL). ALDH1L1-CreER::Ai14 mice specifically labeled Müller glia but not retinal astrocytes (Supplementary Fig. 6a–c). We found that both Müller glia (visualized by tdTomato) and retinal astrocytes (visualized by GFAP) were capable of removing the debris of retinal microglia upon CSF1R inhibition (Fig. 1b–d and Supplementary Fig. 3). Therefore, the microglial debris in the spinal cord is removed by astrocytes, whereas that in the retina is removed by Müller glia and astrocytes.

To further confirm the astrocytic engulfment of microglial debris in vitro, we prepared microglial debris by repeated cycles of freezing and thawing in liquid nitrogen to obtain dead microglia. The cell corpses were then labeled with pHrodo, a fluorescent staining dye, at 37 °C for 10 min After that, the pHrodo-labeled debris was incubated with purified astrocytes in complete medium (Supplementary Fig. 7) for 24–72 h (Fig. 1e). The pHrodo-labeled microglial debris was internalized in cultured astrocytes (Fig. 1f–g), and accumulation of microglial debris was detected from 24 to 72 h (Fig. 1g). The net amount of astrocyte-containing debris equals the phagocytosed debris minus the degraded debris. Because excessive amounts of microglial debris were added to the in vitro cell culture system, debris accumulation indicates that the speed of phagocytosis is faster than the speed of intracellular degradation. Thus, microglial debris accumulated in astrocytes. Collectively, this evidence from both in vivo and in vitro experiments demonstrates that astrocytes are able to engulf microglial debris.

**Fig. 2:**
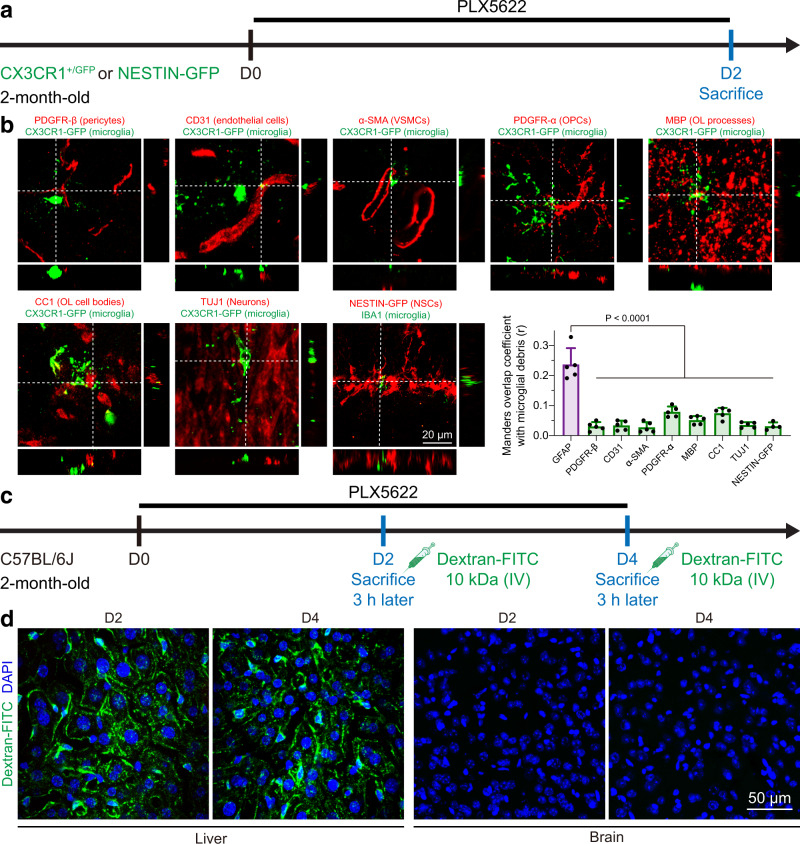
Microglial debris is unlikely to be engulfed by pericytes, endothelial cells, VSMCs, OPCs, oligodendrocytes, NSCs, neurons or circulating blood cells in vivo. **a** Scheme of in vivo debris engulfment examinations by microglial depletion. **b** Microglial debris is unlikely to be phagocytosed by pericytes, endothelial cells, VSMCs, OPCs, oligodendrocytes, neurons or NSCs in vivo. NESTIN-GFP is illustrated by the red pseudocolor for better visualization. Astrocytes (GFAP): 0.24 ± 0.05%, pericytes (PDGFR-β): 0.03 ± 0.01%, endothelial cells (CD31): 0.03 ± 0.01%, VSMCs (α-SMA): 0.03 ± 0.02%, OPCs (PDGFR-α): 0.08 ± 0.02%, oligodendrocytes (MBP): 0.05 ± 0.01%, oligodendrocytes (CC1): 0.07 ± 0.02%, neurons (TUJ1): 0.04 ± 0.01%, NSCs (NESTIN-GFP): 0.03 ± 0.01%. *N* = 4 mice in the NESTIN-GFP group and *N* = 5 mice in the rest of the groups. One-way ANOVA with Holm‒Sidak’s multiple comparisons test (post hoc). *P* = 3.1086e–015, 4.6629e–015, 2.6645e–015, 1.0612e–012, 3.4639e–014, 9.7167e–013, 4.9960e–015, and 1.2434e–014. **c** Scheme of the in vivo BBB integrity examination with 10-kDa dextran-FITC. **d** Confocal images show that the BBB is not compromised during microglial depletion in vivo. Each experiment was independently repeated from 4 mice with similar results. PLX5622: PLX5622-formulated AIN-76A diet; VSMCs vascular smooth muscle cells, OPC oligodendrocyte precursor cell, OL oligodendrocyte, NSC neural stem cell, IV intravenous. The source data are provided as a Source Data file.

In contrast, GFP^+^ microglial debris was barely detected in pericytes (PDGFR-β), endothelial cells (CD31), VSMCs (α-SMA), oligodendrocytes (MBP and CC1) or neurons (TUJ1) (Fig. 2a, b). Although some OPCs (PDGFR-α) were able to phagocytose microglial debris, the engulfed debris content was limited (Fig. 2a, b). Furthermore, by using NESTIN-GFP transgenic mice (aka *Nes*-GFP mice), in which NSCs express GFP^41^, we found that NSCs did not engulf microglial debris (Fig. 2b). In addition, we did not detect a compromised blood‒brain barrier (BBB) on days 2 and 4 after microglia depletion via 10-kDa dextran (Fig. 2c, d), which indicated that microglial debris is unlikely to be engulfed by infiltrating blood cells (e.g., monocytes).

Together, our data indicate that upon microglial ablation, microglial debris is primarily scavenged by astrocytes and retinal Müller glia rather than pericytes, endothelial cells, VSMCs, OPCs, oligodendrocytes, NSCs, neurons or infiltrating blood cells.

### The astrocytic engulfment of microglial debris orchestrates the microglial turnover rate in vivo

**Fig. 3:**
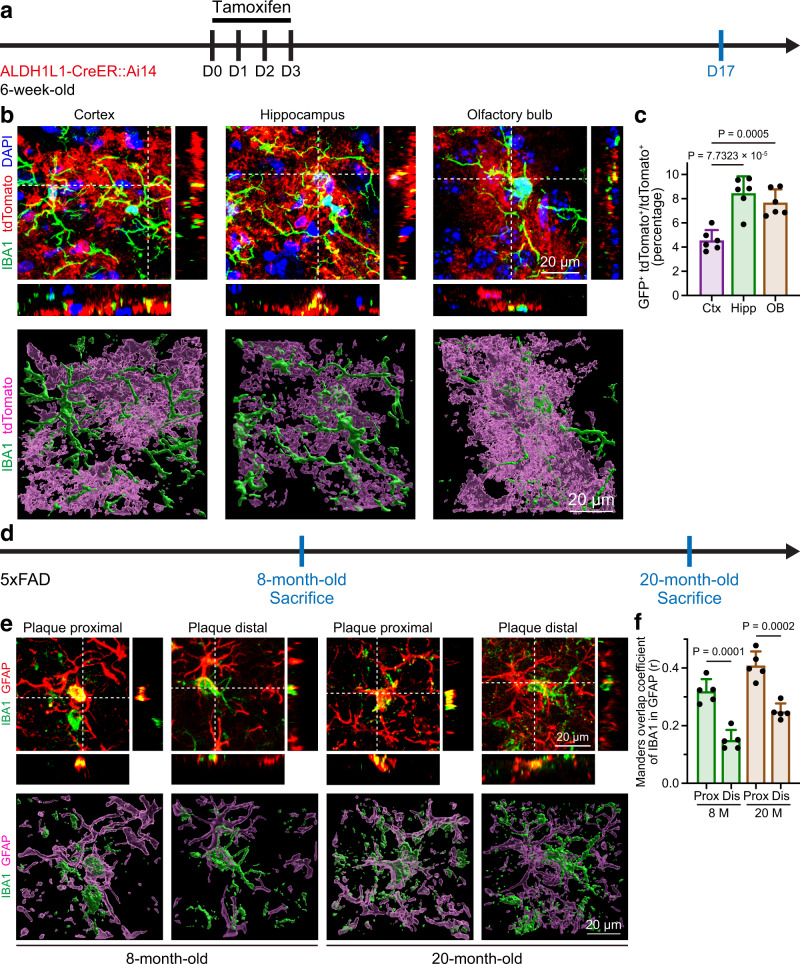
The astrocytic engulfment of microglial debris is positively correlated with the speed of microglial turnover under both physiological and pathological conditions in vivo. **a** Scheme of the in vivo examination of astrocytic engulfment in different brain regions of ALDH1L1-CreER::Ai14 mice under physiological conditions. **b**, **c** Astrocytes in the cortex engulfed less microglial debris than those in the hippocampus and olfactory bulb. *N* = 6 mice for each group. One-way ANOVA with Holm‒Sidak’s multiple comparisons test (post hoc). The quantitative results (**c**) show the results from the reanalysis of the same data from the D19 group in Fig. 1d. **d** Scheme of the in vivo examination of astrocytic engulfment under pathological conditions in 5xFAD mice at 8 and 20 months of age. **e**, **f** Astrocytes in the plaque proximal regions phagocytose more microglial debris than those in the plaque distal regions. *N* = 5 mice for each group. Two-tailed independent *t* test. Ctx cortex; Hipp hippocampus, OB olfactory bulb, Prox plaque proximal, Dis plaque distal. Data are presented as mean ± SD. The source data are provided as a Source Data file.

Whether astrocytic engulfment is relevant to biological functions or occurs in forced microglial depletion is unclear. If astrocytic engulfment plays vital roles in the maintenance of CNS homeostasis, it should be positively correlated with the speed of microglial turnover. We thus explored the biological significance of astrocytic engulfment under two scenarios. First, microglia are heterogeneous cells with variable turnover rates in different brain regions under physiological conditions^42,43^. We thus examined the astrocytic engulfment of microglial debris under physiological conditions in brain regions with different microglial turnover speeds, including the cortex (relatively slow), hippocampus (relatively fast) and olfactory bulb (relatively fast)^24^. Consistent with the regional differences in microglial turnover speeds, the astrocytes in the cortex possessed a relatively low level of GFP^+^ debris, whereas the levels of internalized microglial debris in the hippocampus and olfactory blub astrocytes were relatively high (Fig. 3a–c and Supplementary Fig. 8). Second, microglial turnover is accelerated in plaque-associated brain regions in Alzheimer’s disease (AD)^23^. We then tested the astrocytic engulfment of microglial debris in 5xFAD mice, a mouse model of AD, at 8 and 20 months of age (Fig. 3d) and found that astrocytes in plaque proximal regions exhibited higher capacities for microglial debris engulfment than those in plaque distal regions (Fig. 3e–f).

Therefore, astrocytic engulfment orchestrates microglial turnover under physiological and pathological conditions, which indicates its biological relevance and suggests a critical role in the maintenance of CNS homeostasis.

### Microglia retain the capability of phagocytosing microglial debris in vitro, but territory-dependent competition hinders microglia-to-microglial debris engulfment in vivo

**Fig. 4:**
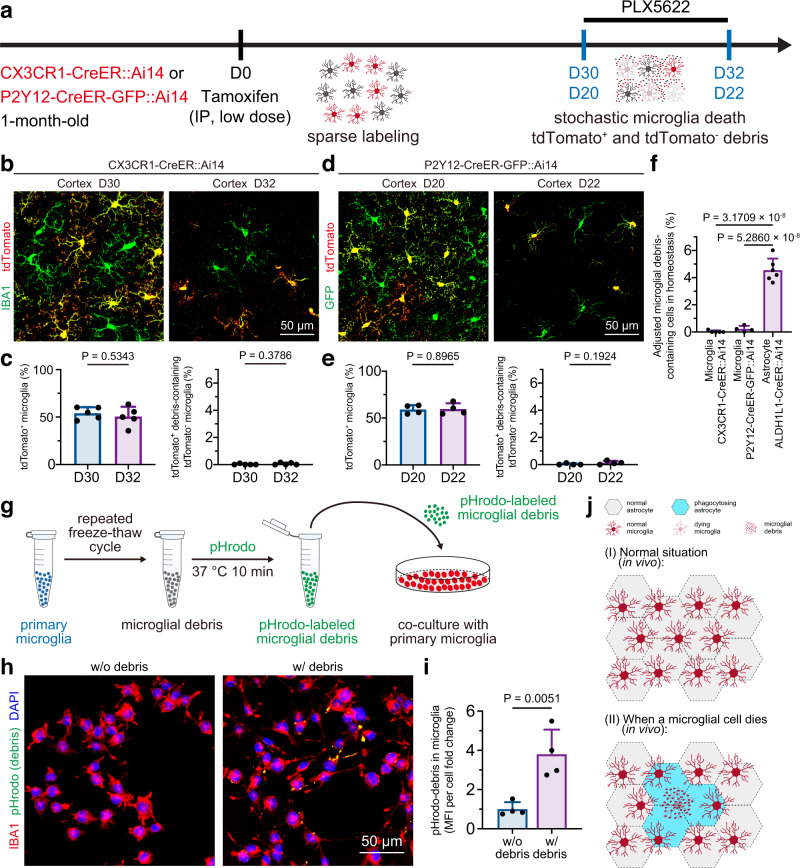
Although microglia are able to engulf microglial debris in vitro, territory-dependent competition hinders microglia-to-microglial debris engulfment in vivo. **a** Scheme of tamoxifen-triggered sparse labeling of microglia for investigation of the microglial engulfment capacity of microglial debris in CX3CR1-CreER::Ai14 and P2Y12-CreER-GFP::Ai14 mice. **b** tdTomato^-^ microglia do not engulf tdTomato^+^ microglial debris under physiological conditions (D30) or upon CSF1R inhibition (D32) in CX3CR1-CreER::Ai14 mice. **c** Quantification of the tdTomato^+^ microglial percentage in CX3CR1-CreER::Ai14 mice. *N* = 5 mice for each group. Two-tailed independent *t* test. **d** tdTomato^-^ microglia do not engulf tdTomato^+^ microglial debris under physiological conditions (D20) or upon CSF1R inhibition (D22) in P2Y12-CreER-GFP::Ai14 mice. **e** Quantification of the tdTomato^+^ microglial percentage in P2Y12-CreER-GFP::Ai14 mice. *N* = 4 mice for each group. Two-tailed independent *t* test. **f** Quantification of tdTomato^+^ microglial debris engulfment by microglia and astrocytes under physiological conditions. The data from the microglial group were adjusted based on the sparse labeling percentage at D30 (CX3CR1-CreER::Ai14) and D20 (P2Y12-CreER-GFP::A14). The data from the astrocytes are obtained from the cortex results shown in Fig. 3c. *N* = 5 CX3CR1-CreER::Ai14 mice, *N* = 4 P2Y12-CreER-GFP::Ai14 mice and *N* = 6 ALDH1L1-CreER::Ai14 mice. One-way ANOVA with Holm‒Sidak’s multiple comparisons test (post hoc). **g** Scheme of the in vitro assay of microglia-to-microglial debris engulfment using pHrodo-labeled microglial debris. **h** IBA1^+^ microglia engulf pHrodo-labeled microglial debris in the cell culture. pHrodo is illustrated by the green pseudocolor for better visualization. **i** Quantification of the microglia-to-microglial debris engulfment after exposure to pHrodo-labeled microglial debris for 24 h. *N* = 4 biological replicates of each group. Two-tailed independent *t* test. **j** The territory-dependent competition model (hypothesis) explains that microglia do not phagocytose microglial debris in vivo. (I) Under normal conditions and (II) when a microglial cell dies. PLX5622: PLX5622-formulated AIN-76A diet; IP intraperitoneal. Data are presented as mean ± SD. The source data are provided as a Source Data file.

Microglia are professional phagocytes in the CNS^1,2^. We thus further asked whether microglia are able to remove their congeneric cell debris. The major obstacle to studying microglia-to-microglia engulfment is distinguishing engulfed microglial debris from engulfed microglia in vivo. To this end, we designed a sparse labeling system by treating CX3CR1-CreER::Ai14 mice with a low dose of tamoxifen (Fig. 4a). Thirty days after tamoxifen treatment, approximately 54% of microglia were labeled with tdTomato (Fig. 4a–c), and the animals were then administered PLX5622 to partially deplete microglia (Fig. 4a). Two days after depletion, approximately 51% of microglia were tdTomato^+^, and this value is comparable to the percentage before PLX5622 treatment (Fig. 4c). The results indicate that tdTomato^+^ and tdTomato^−^ microglia are identically ablated upon PLX5622 treatment. If surviving microglia are capable of engulfing microglial debris (e.g., tdTomato^−^ microglia engulf tdTomato^+^ debris), tdTomato^+^ debris should be observed in tdTomato^−^ microglia. However, tdTomato^+^ debris was not detected in tdTomato^−^ microglia (Fig. 4b, c), which suggests that the surviving microglia do not phagocytose microglial debris. On the one hand, CX3CR1-CreER::Ai14 (*Cx3cr1*^*wt/ko-CreER*^*;Ai14*^*wt/mut*^) mice are *Cx3cr1*-haploinsufficient. On some occasions, *Cx3cr1*-haploinsufficient microglia might exhibit a nonidentical phenotype to wild-type microglia^43^. To avoid its potential influence and more rigidly confirm this conclusion, we generated P2Y12-CreER-GFP mice through in situ insertion of P2A-CreER^T2^-P2A-GFP between the last exon and the 3ʹ-UTR of the *P2ry12* gene (Supplementary Fig. 9a). Almost all microglia expressed GFP, whereas non-myeloid cells in the brain were negative for GFP (Supplementary Fig. 9b-c). We thus sparsely labeled microglia in P2Y12-CreER-GFP::Ai14 mice and ablated microglia with PLX5622 (Fig. 4a). Similarly, tdTomato^+^ debris was still not detected in tdTomato^−^ microglia of P2Y12-CreER-GFP::Ai14 mice (Fig. 4d, e), which further confirmed that the surviving microglia do not phagocytose microglial debris. On the other hand, the surviving microglia might be dysfunctional upon PLX5622 administration, which may not reflect the debris engulfment capability under physiological conditions. To address this question, we also compared the engulfment of debris under physiological conditions between sparsely labeled CX3CR1-CreER::Ai14 and P2Y12-CreER-GFP::Ai14 mice with ALDH1L1-CreER::Ai14 mice before CSF1R inhibition (Figs. 4a and 3a). Even under physiological conditions (with a relatively low microglial turnover rate), microglia debris-containing astrocytes were observed (Fig. 4f, 4.552%). In contrast, no obvious tdTomato^+^ debris was detected in tdTomato^−^ nondysfunctional microglia (Fig. 4f, 0.036% in CX3CR1-CreER::Ai14 mice and 0.229% in P2Y12-CreER-GFP::Ai14 mice, adjusted by the sparse labeling efficiency). Therefore, our results indicate that microglial debris is not primarily removed by microglia. Notably, compared with the microglial debris engulfment rate of microglia (almost zero), the astrocytic engulfment rate under physiological conditions (Fig. 4f) further confirmed its physiological relevance in microglial debris removal.

Next, we asked why microglia, the primary scavenger of the CNS, do not phagocytose their congeneric debris in the brain. We thus tested the capability of microglia-to-microglial debris phagocytosis in vitro. Primary microglia were harvested^36,44^ and cocultured with pHrodo-labeled microglial debris for 24 h (Fig. 4g). Unexpectedly, the pHrodo-labeled microglial debris was phagocytosed by the cultured microglia (Fig. 4h, i), which suggested that microglia retain the capability of engulfing their congeneric debris. Provided that our in vivo sparse labeling did not observe obvious microglia-to-microglial debris phagocytosis in physiological condition or upon microglial depletion (Fig. 4a–f), we thus concluded a territory-dependent competition model (hypothesis): microglia and astrocytes competitively phagocytose microglial debris. Under normal conditions, microglia evenly tile the brain and do not invade the territory of their congeneric neighbors (Fig. 4j (I)). In contrast, the territories between astrocytes and microglia overlapped (Fig. 4j (I)). When a microglial cell dies, the cell locally collapses (Fig. 4j (II)). The territory-overlapping astrocytes are close to dead microglia (Fig. 4j (II); cyan hexagons), whereas neighboring microglia are relatively distal (Fig. 4j (II); red microglia). Microglial debris is first removed by overlapping astrocytes before spreading to distal microglia for phagocytosis. Therefore, even though microglia can engulf their congeneric debris, microglial debris is competitively removed by spatial proximal astrocytes before coming into contact with relatively distal microglia. As a result, microglial debris is primarily removed in vivo by astrocytes rather than microglia. This territory-dependent competition model echoes previous observations of olfactory nerve debris clearance by olfactory ensheathing cells, in which little phagocytosis by distal microglia/macrophages was detected^45^.

### Microglial debris is not removed by CNS border-associated macrophages in vivo

**Fig. 5:**
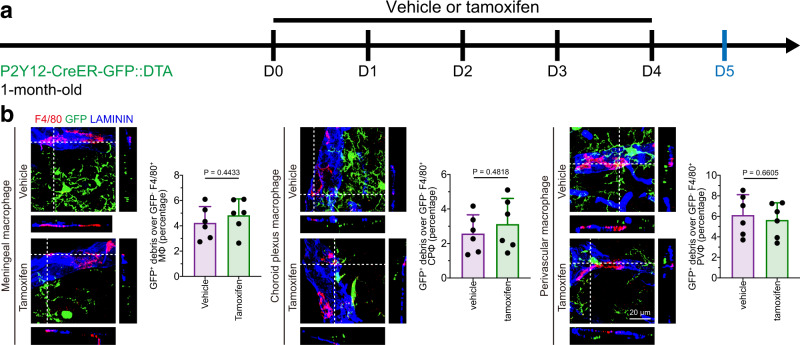
BAMs do not engulf microglial debris in vivo. **a** Scheme of tamoxifen-triggered microglial cell death for investigating the BAM engulfment capacity of microglial debris. **b** Meningeal, choroid plexus or perivascular macrophages do not engulf parenchymal microglia upon microglial ablation. *N* = 6 mice in each group. Two-tailed independent *t* test. MΦ meningeal macrophage, CPΦ choroid plexus macrophage, PVΦ perivascular macrophage. Data are presented as mean ± SD. The source data are provided as a Source Data file.

Brain myeloid cells comprise microglia and BAMs. Our P2Y12-CreER-GFP mouse can distinguish parenchymal microglia (GFP^+^) from meningeal, choroid plexus and perivascular macrophages (GFP^-^) (Supplementary Fig. 9b, c). To test whether BAMs are able to phagocytose microglial debris, we utilized tamoxifen to induce microglial cell death without affecting BAMs in P2Y12-CreER-GFP::DTA mice (Fig. 5a). Five days after tamoxifen administration, 30.10% of microglia were ablated. However, meningeal, choroid plexus or perivascular macrophages (F4/80^+^ GFP^−^) did not engulf GFP^+^ debris upon microglial depletion (Fig. 5b). Therefore, microglial debris is not removed by BAMs.

### Microglial debris-phagocytosing astrocytes exhibit a noncanonical reactive phenotype in vivo

**Fig. 6:**
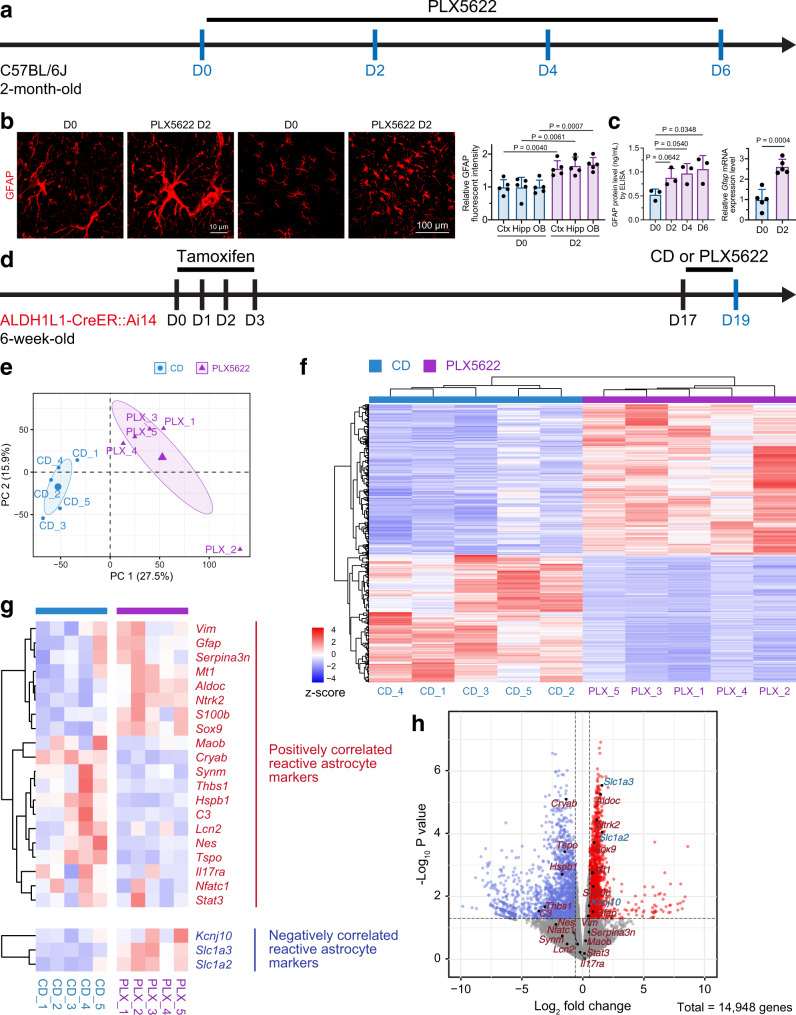
Astrocytes exhibit a noncanonical and sophisticated reactive phenotype when engulfing microglial debris in vivo. **a** Scheme of in vivo microglial depletion and time points for immunostaining, ELISA and qPCR analysis. GFAP is upregulated in astrocytes after microglial depletion in vivo, as shown by immunostaining (**b**), ELISA (**c**) and qPCR (**c**). *N* = 5 (immunostaining and qPCR) and 3 mice (ELISA) in each group. Two-tailed independent *t* test (immunostaining and qPCR) and one-way ANOVA with Holm‒Sidak’s multiple comparisons test (post hoc) (ELISA). **d** Scheme of in vivo microglial depletion and time points for RNA-seq analysis. **e** PCA of astrocytes. **f** Heatmap of differentially expressed genes in PLX5622-treated microglia. **g** Heatmap of reactive astrocyte markers in the reactive astrocyte consensus statement^47^. **h** Volcano plot of reactive astrocyte markers in the reactive astrocyte consensus statement^47^. Red dots: upregulated differentially expressed genes; blue dots: downregulated differentially expressed genes; genes in red: positively correlated reactive astrocyte markers; genes in blue: negatively correlated reactive astrocyte markers. Quasi-likelihood (QL) F tests were used for statistical testing. Genes with *P* < 0.05 and |log_2_(fold change)| > log2(1.5) were determined to be statistically significant. *N* = 5 mice in each group (**d**–**h**). PLX5622 PLX5622-formulated AIN-76A diet, CD control AIN-76A diet. Data are presented as mean ± SD. The source data are provided as a Source Data file.

By immunostaining (in vivo), enzyme-linked immunosorbent analysis (ELISA; magnetic-activated cell sorting (MACS)-sorted astrocytes) and quantitative PCR (qPCR; MACS-sorted astrocytes), we observed robust upregulation of GFAP in astrocytes during microglial depletion (Fig. 6a–c). We and other research groups previously demonstrated that microglial depletion does not induce inflammatory responses^27,28,30,31^; thus, astrocytes are not directly influenced by inflammatory microenvironments upon PLX5622 treatment. In addition, astrocytes do not express CSF1R and thus cannot be directly influenced by PLX5622. Therefore, the alteration of astrocytes from PLX5622-treated mice represents the response of astrocytes to the phagocytosis of microglial debris. The upregulation of GFAP in PLX5622-treated mice suggests that astrocytes exhibit a reactive-like phenotype during microglial debris clearance.

To understand how astrocytes respond to microglial debris engulfment, we utilized fluorescence-activated cell sorting (FACS) to harvest tdTomato^+^ astrocytes from tamoxifen-treated ALDH1L1-CreER::Ai14 mice^38^ and compared the gene profiles upon CD and PLX5622 administration by RNA sequencing (RNA-seq) (Fig. 6d; gating strategy in Supplementary Fig. 10a). The RNA-seq data were validated by qPCR (Supplementary Fig. 10b-c). Principal component analysis (PCA) revealed that the transcriptome characteristics of normal astrocytes (CD group) were different from those of astrocytes upon PLX5622 administration (Fig. 6e). During microglial debris engulfment, 542 genes were upregulated, whereas 459 genes were downregulated (definition of differentially expressed genes: |fold change | >= 2 and FDR < = 0.01) (Fig. 6f). We then investigated the reactive astrocyte markers of the reactive astrocyte consensus statement^46^. Most of the reactive astrocyte markers tended to exhibit differential expression (Fig. 6g, h). Among the positively correlated reactive astrocyte markers^46^, only 8 out of 20 genes were upregulated (Fig. 6g, h), and 12 out of 20 genes were downregulated or not significantly changed (Fig. 6g, h). In contrast, among the negatively correlated reactive astrocyte markers^46^, an upregulation trend was observed (Fig. 6g, h). Therefore, the RNA-seq results suggest that astrocytes exhibit a noncanonical and sophisticated reactive phenotype when engulfing microglial debris.

### Microglial debris engulfment is facilitated by C4b opsonization in vivo and in vitro

**Fig. 7:**
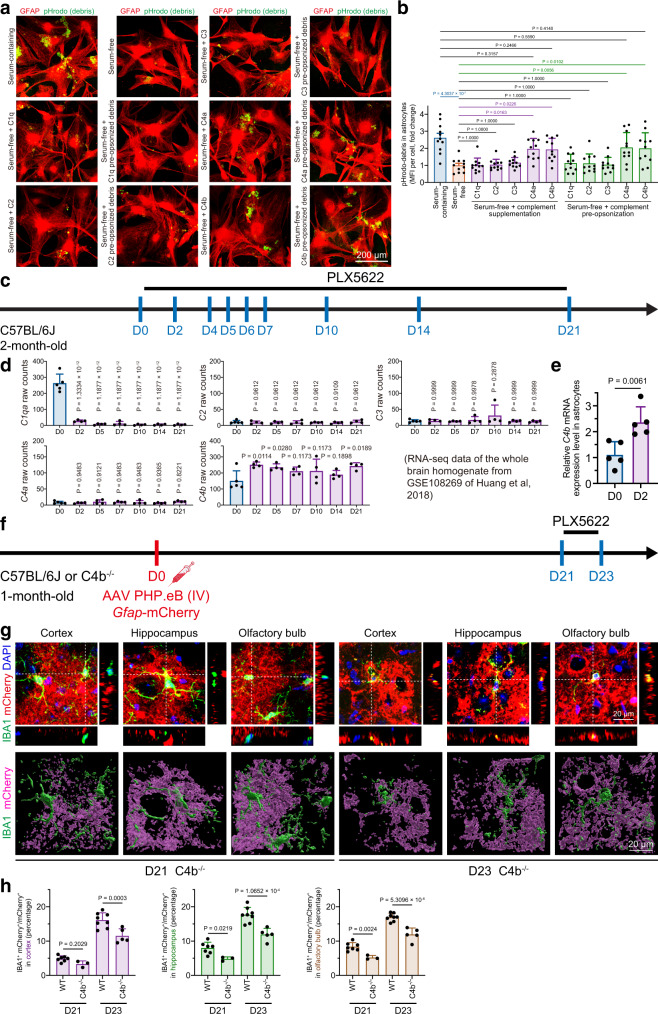
C4a and C4b can facilitate the astrocytic engulfment of microglial debris in vivo and in vitro. **a** C4a and C4b restore the astrocytic engulfment of microglial debris in vitro in serum-free culture medium. **b** Quantifications of the phagocytic influence by complement supplementation and preopsonization in serum-free culture medium. *N* = 11 independent biological replicates of each group. One-way ANOVA with Holm‒Sidak’s multiple comparisons test (post hoc). **c** Scheme of in vivo microglial depletion and time points for analysis. **d** Reanalysis of RNA-seq data from whole-brain homogenate (GSE108269^27^) showing that *Gfap* and *C4b* are upregulated and *C1qa* is downregulated during microglial ablation, whereas *C2*, *C3* and *C4a* remain at low levels and are unaffected. *N* = 5 mice at D0 and *N* = 4 mice at D2 to D21. One-way ANOVA with Holm‒Sidak’s multiple comparisons test (post hoc). **e** qPCR further confirmed the upregulation of *C4b* in sorted astrocytes upon microglial depletion. *N* = 5 in each group. Two-tailed independent *t* test. **f** Scheme of the in vivo examination of astrocytic engulfment using AAV PHP.eB-based astrocyte labeling and microglial depletion. **g** Confocal orthogonal colocalization and 3D reconstruction show that C4b^−/−^ impairs the astrocytic engulfment of microglial debris under physiological condition (D21) and upon CSF1R inhibition (D23). **h** Quantification of microglial debris engulfment by astrocytes. *N* = 7 (D21) and 8 (D23) WT mice, and *N* = 3 (D21) and 5 (D23) C4b^−/−^ mice. One-way ANOVA with Holm‒Sidak’s multiple comparisons test (post hoc). PLX5622 PLX5622-formulated AIN-76A diet, CD control AIN-76A diet, IV intravenous, MFI mean fluorescence intensity, Ctx cortex, Hipp hippocampus, OB olfactory bulb. Data are presented as mean ± SD. The source data are provided as a Source Data file.

We asked how astrocytes engulf microglial debris. Complement components play key roles in the phagocytosis process^47^; thus, we first examined whether complement opsonization mediates astrocytic engulfment of microglial debris. Compared with the results obtained using culture medium with complement component-containing serum (FBS), the engulfment of microglial debris by astrocytes cultured in serum-free medium (without FBS) was significantly suppressed (Fig. 7a, b). We subsequently tested the potential role of C1q, C2, C3, C4a and C4b, the major complement components in the CNS, by both supplementation and preopsonization. We found that C4a and C4b were able to restore the engulfment capability of astrocytes, whereas C1q, C2 or C3 were not (Fig. 7a, b). Astrocytes increase their cellular processes in serum-free medium over time^48,49^, as we showed that astrocytes cultured for an extended period of 14 days in serum-free medium display a significant number of processes (Supplementary Fig. 11a), mimicking the astrocyte morphology in vivo. We further confirmed that in serum-free astrocytes cultured for 14 days, C4b still facilitates the astrocytic engulfment of microglial debris (Supplementary Fig. 11b). Similar results were detected in the serum-free culture medium (Supplementary Fig. 12a, b). In addition, we found that microglial debris colocalized with C4 both in vivo and in vitro (Supplementary Fig. 12c–e), which indicated that microglial debris is opsonized by C4 upon microglial death and fragmentation.

Next, we reanalyzed the expression levels of *C1qa*, *C2*, *C3*, *C4a* and *C4b* in microglia-depleted brains using our previously published RNA sequencing (RNA-seq) database of bulk tissue from whole brain homogenates^27^. We found that *C2*, *C3* and *C4a* were expressed at relatively low levels in the brain and that the levels did not changed upon microglial ablation (Fig. 7c, d). Although C4a is able to facilitate debris engulfment in vitro, it is unlikely to play a major role in the brain due to its low expression level. In contrast, we observed a significant upregulation of *C4b* (Fig. 7c, d), the opsonin that facilitates cell debris clearance by professional phagocytes^47^. We double confirmed the upregulation of C4b in MACS-sorted astrocytes, one of the major sources, during microglial depletion by qPCR (Fig. 7e). Converging evidence thus suggests that the astrocytic engulfment of microglial debris is facilitated by C4b in vivo. To test this hypothesis in vivo, we generated C4b^−/−^ mice by CRISPR/Cas9-mediated genome editing (Supplementary Fig. 13) and utilized F0 founder mice (either biallelic or multiallelic mosaic), which were verified lacking C4 expression (Supplementary Fig. 14). Compared with the results found for the wild-type, the level of engulfed microglial debris was significantly reduced in C4b^−/−^ astrocytes under physiological conditions (except for the cortex, most likely due to the low turnover rate in this brain region) (Fig. 7f–h; D21) and upon microglial depletion (Fig. 7f–h; D23). Therefore, our results indicate that complement C4b opsonizes microglial debris and facilitates astrocytic engulfment in vivo.

We detected nearly complete ablation of *C1qa* (Fig. 7c, d), *C1qb* and *C1qc* (refer to GSE108269^27^) during microglial depletion. Microglia are the major source of C1q^50^; therefore, this downregulation should be attributed to massive microglial depletion. Because C1q facilitates the phagocytosis process as an “eat-me” signal^7,51,52^, its downregulation during microglial depletion suggests that C1q does not participate in microglial debris clearance. To fully confirm this conclusion in vivo, we examined the engulfment of microglial debris in C1qa^−/−^ mice (Supplementary Fig. 15a). We found that C1q deficiency did not affect the astrocytic engulfment of microglial debris under physiological conditions or upon microglial depletion (Supplementary Fig. 15b-c), confirming the C1q-independent mechanism. This finding also indicates that C4-mediated microglial removal is not a compensation of C1q absence.

### Intra-astrocytic microglial debris is degraded via LAP in vivo and in vitro

**Fig. 8:**
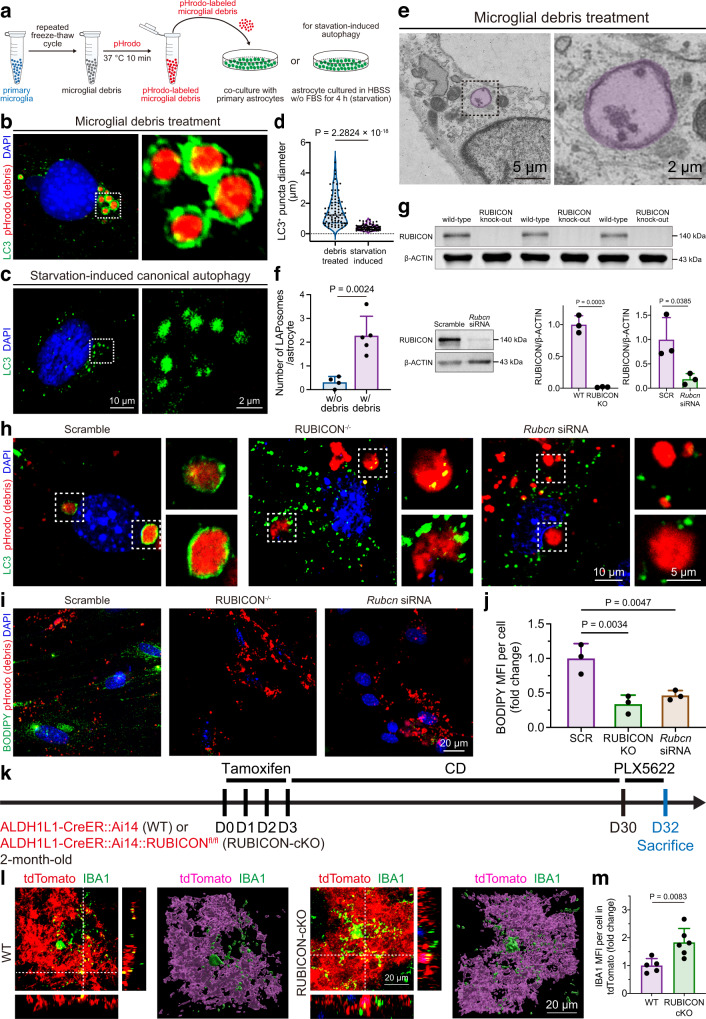
Engulfed microglial debris is degraded by LAP in vivo and in vitro. **a** Schemes of the in vitro astrocytic engulfment assay using pHrodo-labeled microglial debris and starvation-induced autophagy. Astrocytic LAPosomes in microglial debris engulfment (**b**) and autophagosomes in starvation-induced autophagy (**c**) in vitro. **d** Quantification of LAPosome and autophagosome diameters in vitro. *N* = 81 phagosomes from 4 to 5 mice of each group. Two-tailed independent *t* test. **e** Electron microscopy of microglial debris-containing LAPosomes in astrocytes cultured with debris. **f** Quantification of LAPosomes in astrocytes in vitro by electron microscopy. *N* = 4 (without debris) and 5 (with debris) mice of each group. Two-tailed independent *t* test. **g** Validation of RUBICON knockout and knockdown in primary cultured astrocytes by Western blotting. *N* = 3 independent biological replicates of each group. Two-tailed independent *t* test. **h** *Rubcn* knockout or knockdown prevents the formation of microglial debris-containing LAPosomes in vitro. The puncta of LC3^+^ autophagosomes are significantly increased in these knockout or knockdown cells. Each experiment is independently repeated using 4 mice with similar results. **i**, **j** RUBICON knockout or knockdown reduces the degradative product (neutral lipids) in astrocytes in vitro. *N* = 3 biological replicates of each group. Two-tailed independent *t* test. **k** Schemes of studying the in vivo consequences of RUBICON conditional knockout in astrocytes on the degradation of engulfed microglial debris in astrocytes. **l**, **m** RUBICON conditional knockout in astrocytes in vivo dampens the degradation of engulfed microglial debris and thus results in the accumulation of nondegraded debris in astrocytes. *N* = 5 and 6 ALDH1L1-CreER::Ai14 (WT) mice and ALDH1L1-CreER::Ai14::RUBICON^fl/fl^ (RUBICON-cKO) mice, respectively. Two-tailed independent *t* test. WT wild-type, SCR scramble; KO RUBICON knockout, KD RUBICON knockdown, PLX5622 PLX5622-formulated AIN-76A diet, CD control AIN-76A diet, MFI mean fluorescence intensity. Data are presented as mean ± SD (bar plot) or median ± quartile (violin plot). The source data are provided as a Source Data file.

We subsequently explored the mechanism through which the engulfed microglial debris is degraded in astrocytes. We found that genes that were differentially expressed in microglia-depleted brains were enriched in autophagy among the biological process terms, as determined by the Gene Ontology (GO) analysis (gene ratio: 13/371, Q value: 0.0287). The autophagy machinery was recently found to be coupled with phagocytosis, in which LC3 associates with phagosomes to facilitate phagosome fusion with lysosomes and enhance the degradative efficiency, and this noncanonical autophagy process is termed LAP^53,54^. We found that LC3 is associated with intra-astrocytic microglial particles (Fig. 8a, b), which suggests that the internalized debris is degraded through autophagy. The diameter of autophagosomes in canonical autophagy is typically 0.5–1 μm^55,56^. In contrast, the LAPosomes in LAP, a noncanonical autophagy, typically have diameters larger than 1 μm^56^. Based on our observations, the diameter of intra-astrocytic microglial fragment-containing LC3^+^ organelles was 1.52 ± 0.98 μm, significantly larger than the diameter of autophagosomes (0.42 ± 0.18 μm) in starvation-induced canonical autophagy (Fig. 8b–d). This finding indicates that microglial debris is degraded through the LAP mechanism. We further characterized the membrane ultrastructure by transmission electron microscopy (TEM). Different from the double-membrane autophagosome of canonical autophagy, the LAPosomes of noncanonical autophagy form a single membrane^53,56,57^. Our results showed that the debris-containing single-membrane structures (LAPosomes of noncanonical autophagy) were markedly increased in microglial debris-engulfing astrocytes (Fig. 8e–f), which further confirmed the noncanonical autophagy mechanism. Another discrepancy between canonical and noncanonical autophagy is the molecular mechanism governing these two processes. Despite sharing some core machineries with canonical autophagy, LAP is uniquely dependent on RUBICON^54,56^, a negative regulator of canonical autophagy^58,59^. When RUBICON is knocked out or knocked down (Fig. 8g), astrocytes failed to form LAPosomes encapsulating the microglial debris but showed increased autophagosome puncta formation (Fig. 8h). As a consequence, the nondegraded microglial debris accumulated in RUBICON-knockout (Fig. 8k–m, Supplementary Fig. 16a–d) or RUBICON-knockdown astrocytes (Supplementary Fig. 16e–h). In addition, the levels of neutral lipids, the degradative product of cell debris^26,56^, were significantly reduced in RUBICON-knockout or RUBICON-knockdown astrocytes (Fig. 8i–j). Therefore, the intra-astrocytic debris is degraded via RUBICON-dependent LAP.

**Fig. 9:**
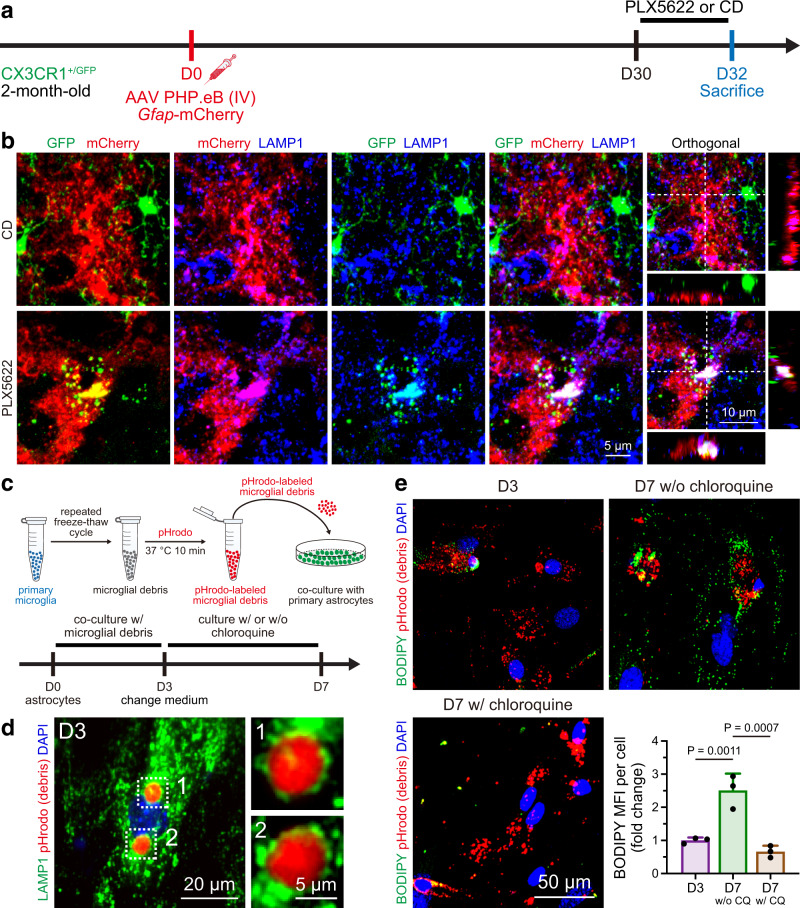
Microglial debris-containing LAPosomes are fused with acidic lysosomes for degradation in vivo and in vitro. **a** Scheme of the in vivo examination of astrocytic engulfment involving AAV PHP.eB-based astrocyte labeling and microglial depletion. **b** Intra-astrocytic microglial debris colocalized with LAMP1 in vivo. Each experiment was independently repeated from 7 mice with similar results. **c** Schemes of the in vitro astrocytic engulfment assay using pHrodo-labeled microglial debris. **d** pHrodo-labeled microglial debris localized with LAMP1 in in vitro cultured astrocytes. Each experiment was independently repeated from 4 biological replicates with similar results. **e** The degradative product (lipid droplets) is reduced after disruption of the formation of phagolysosomes in vitro by chloroquine. D3: 1.00 ± 0.09, D7 w/o CQ: 2.51 ± 0.51, D7 w/ CQ: 0.66 ± 0.18. *N* = 3 biological replicates of each group. One-way ANOVA with Holm‒Sidak’s multiple comparisons test (post hoc). PLX5622: PLX5622-formulated AIN-76A diet; CD control AIN-76A diet, IV intravenous. Data are presented as mean ± SD. The source data are provided as a Source Data file.

The intra-astrocytic microglial fragments colocalized with the lysosomal marker LAMP1 in PLX5622-treated mice (Fig. 9a, b) and primary astrocyte cultures (Fig. 9c, d). In addition, both GO and Kyoto Encyclopedia of Genes and Genomes (KEGG) analyses revealed that “lysosome” was enriched in astrocytes of the microglial depletion brain at day 2 (GO: gene ratio: 49/969, Q value: 1.47 × 10^−8^; KEGG: 20/481, Q value: 1.81 × 10^−3^). These results suggest that the debris-containing LAPosomes fused with lysosomes to form phagolysosomes for debris degradation. Indeed, when chloroquine was used to interfere with the formation of phagolysosomes^56,60^, the level of neutral lipids, which are the degradation product, was significantly reduced in astrocytes (Fig. 9e). Thus, microglial debris-containing LAPosomes fuse with acidic lysosomes to form phagolysosomes for further degradation.

Collectively, our results demonstrate that phagocytosed microglial debris is degraded through the LAPosome-to-phagolysosome axis in astrocytes.

**Fig. 10:**
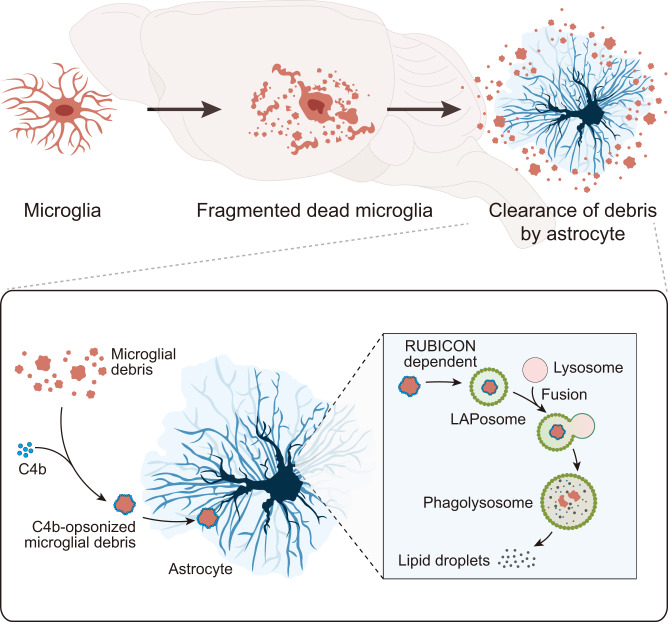
Model of microglial debris removal and degradation by astrocytes in the brain. The debris of microglia (professional phagocytes) is primarily engulfed by astrocytes (nonprofessional phagocytes), which is facilitated by C4 opsonization. The phagocytosed debris is then degraded in astrocytes via RUBICON-dependent LAP, and microglial debris-containing LAPosomes subsequently fuse with lysosomes to form phagolysosomes, in which the debris is degraded.

In summary, microglial debris is primarily engulfed by astrocytes, and this process is facilitated by C4 opsonization. The phagocytosed debris is then degraded in astrocytes via RUBICON-dependent LAP, and microglial debris-containing LAPosomes then fuse with lysosomes to form phagolysosomes, in which the debris is degraded into lipid droplets (Fig. 10).

## Discussion

As professional phagocytes and scavengers in the CNS, microglia are the major players engulfing cell corpses, excessive dendritic spines and Aβ aggregates^3–7,11–14^. However, the identity of the cells that scavenge corpses of the professional scavenger is largely neglected. Importantly, even when microglia are massively and rapidly killed, the cell debris can be removed in a timely manner to avoid cellular debris accumulation^27–30^. These findings imply the biological significance of microglial debris clearance in the maintenance of CNS homeostasis and function, but the cellular and molecular mechanisms remain elusive.

Microglia do not participate in the removal of congeneric microglial debris in vivo (at least not the major scavenger). Furthermore, we excluded the participation of pericytes, endothelial cells, VSMCs, OPCs, oligodendrocytes, NSCs, neurons and infiltrating blood cells. Although the phagocytic capacities of microvascular endothelial cells^26^, OPCs^61,62^ and neural stem (progenitor) cells^63^ have been reported by us and other groups, these cells are not extensively involved in microglial debris clearance.

Instead, we found that microglial debris is primarily removed by astrocytes. Although professional phagocyte microglia can engulf nonprofessional phagocyte astrocytes^15^, the removal of debris from these professional phagocytes is conducted by the nonprofessional phagocytes. Furthermore, we investigated the cellular and molecular mechanisms. Astrocyte engulfment is mediated by the opsonization of C4. The phagocytic cargos are then degraded via LAP, a form of noncanonical autophagy. As glial cells and nonprofessional phagocytes in the CNS, astrocytes reportedly clear the debris of dysfunctional neurons in brain injury and neurodegenerative disorders^32,33^. A recent study demonstrated that astrocytes and microglia coordinate to clear cell corpses: microglia phagocytose cell bodies and apical dendrites of dying neurons, whereas astrocytes engulf numerous small dendritic apoptotic bodies^34^. When *Irf8* deficiency disables microglial phagocytosis, astrocytes can play a complementary role^64^. Although these studies focused on delineating astrocytic phagocytosis, little attention has been given to the astrocytic contribution to microglial turnover, which has been partially attributed to the professional phagocytic role of microglia per se. Therefore, our study reveals the almost neglected yet important role of astrocytes in microglial debris clearance. In addition, although previous studies reported the phagocytic roles of astrocytes^32–34^, the mechanism through which internalized cargos are routed to the degradation machinery remains unclear. Our current findings show how internalized cargos are degraded in astrocytes and thus offer insights into this nonprofessional phagocytic cell.

In the homeostatic brain, the microglial turnover (death) rate is approximately 0.05 to 0.2% per day^20,23,65,66^. In contrast, approximately 15% of cells are ablated within the first 24 h upon PLX5622 administration^27^, and this value is 75 to 150 times faster than that under normal conditions. Even if numerous microglia are ablated within a relatively short period, the cell fragments are not evidently accumulated^27–31,67^. In addition, microglia can respond to neural injury and migrate toward the injury site within 1 h^68,69^. Even with such a rapid migration speed, microglia do not largely engulf their neighboring microglial debris upon territory-dependent competition of astrocytes in vivo. Thus, rapid cellular corpse removal indicates an efficient, even excessive, phagocytic capacity of astrocytes. The excessive astrocytic capability implies the significance of microglial debris removal that guarantees the microenvironment not affected.

In contrast, microglia show accelerated cell death and/or turnover in CNS disorders. For instance, in ischemic stroke, microglia show substantial proliferation at the early stage. The cell number is then steeply reduced due to microglial cell death^70^. In addition, plaqueproximal microglia exhibit a several-fold higher turnover rate than plaque distal microglia in AD^23^. Thus, the excessive phagocytic ability of astrocytes ensures the timely removal of microglial debris, which confines a secondary injury and dampens CNS dysfunction.

Collectively, the excessive capacity of phagocytosis by astrocytes might be critical to protect the CNS from the influence of accumulated cell debris thereby maintaining the CNS homeostasis.

## Methods

### Ethics statements

All mouse experiments were conducted in accordance with the guidelines of the Institutional Animal Care and Use Committee of Fudan University, Shenzhen Institute of Advanced Technology at Chinese Academy of Sciences and Shanghai Mental Health Center at Shanghai Jiao Tong University School of Medicine. This study was approved by the Institutional Animal Care and Use Committee of Shenzhen Institute of Advanced Technology at Chinese Academy of Sciences (SIAT-IACUC-190312-YGS-PB-A0576-01) and the Institutional Animal Care and Use Committee of Fudan University (2021JS ITBR-002).

### Experimental animals

CX3CR1^GFP/GFP^ (B6.129P-Cx3cr1tm1Litt/J, Stock No: 005582)^37^, CX3CR1-CreER (B6.129P2(C)-Cx3cr1tm2.1(cre/ERT2)Jung/J, Stock No: 020940)^71^, Ai14 (B6. Cg-Gt(ROSA)26Sortm14(CAG-tdTomato)Hze/J, Stock No: 007914)^72^, 5xFAD (B6. Cg-Tg(APPSwFlLon,PSEN1*M146L*L286V)6799Vas/Mmjax, Stock No: 34848-JAX)^73^, DTA (B6.129P2-Gt(ROSA)26Sortm1(DTA)Lky/J, aka Rosa26-DTA)^74^, C1qa^−/−^ (B6(Cg)-C1qatm1d(EUCOMM)Wtsi/TennJ)^50^ and RUBICON^−/−^ (C57BL/6-Rubcnem1Dgre/J)^54^ mice were purchased from The Jackson Laboratory. RUBICON^fl/fl^ (B6/JGpt-Rubcnem1Cflox/Gpt) mice were purchased from GemPharmatech. NESTIN-GFP (*Nes*-GFP) mice were procured from Cyagen Biosciences China^41^. C57BL/6 J mice were purchased from Charles River (Beijing Vital River Laboratory Animal Technology). ALDH1L1-CreER mice were kindly donated by Prof. Tian-Ming Gao at Southern Medical University^38^.

C4b^−/−^ mice were generated by CRISPR/Cas9-mediated genome editing. sgRNAs were designed using CHOPCHOP. Two selected sgRNAs targeting exons 1 and 2 of all four *C4b* transcripts, namely, GCTCCTCTGGGGGCTGGCCT and GCTGTACCCCCACCGACAGG were constructed by fusion of a T7 promoter and tracrRNA via overlapping PCR. Full-length sgRNA was produced and transcribed. The transcribed sgRNA and Cas9 mRNA were both purified. The mixture of sgRNA (25 ng/μL each) and Cas9 mRNA (50 ng/μL) was microinjected into the cytoplasm of zygotes of C57BL/6 mice to generate C4b^−/−^ mice. Fifty-five founder mice (F0) were born and analyzed for CRISPR-edited indels. Mouse tail clips were used for PCR amplification with primers flanking the sgRNA regions ACGCACATGCACAGGGACAC and TCAAGGCTGAGCAGCACAAA. The amplicons were directly Sanger sequenced (Supplementary Data 1) and then subjected to CRISPR edit analysis of *C4b* indels using the Synthego ICE CRISPR Analysis Tool (https://www.synthego.com/products/bioinformatics/crispr-analysis). Among the 55 F0 founder lines generated in this study, 8 were identified as *C4b* indel mutants with >83% frameshift indels, including 5 biallelic lines (either homozygotes or compound heterozygotes) and 3 multiallelic mosaics.

P2Y12-CreER-GFP (*P2ry12*-p2A-CreER-p2A-EGFP) mice were generated by Beijing Biocytogen through CRISPR/Cas9-based extreme genome editing (EGE) according to the authors’ design as a “fee-for-service”. The p2A-CreER^T2^-p2A-EGFP cassette was inserted between the last exon of the *P2ry12* gene (transcript 201), the gene encoding P2Y12, and its 3ʹ UTR.

ALDH1L1-CreER mice were crossed with Ai14 mice to obtain ALDH1L1-CreER::Ai14 (ALDH1L1^+/CreER^::Ai14^wt/mut^) mice. ALDH1L1-CreER mice were crossed with Ai14 and RUBICON^fl/fl^ mice to obtain ALDH1L1-CreER::Ai14::RUBICON^fl/fl^ (ALDH1L1-CreER^+/CreER^::Ai14 ^wt/mut^::RUBICON^fl/fl^) mice, aka RUBICON-cKO mice. CX3CR1-CreER mice were crossed with Ai14 mice to obtain CX3CR1-CreER::Ai14 (CX3CR1^+/CreER^::Ai14^wt/mut^) mice. P2Y12-CreER-GFP mice were crossed with Ai14 mice to obtain P2Y12-CreER-GFP::Ai14 (P2Y12^+/CreER-GFP^::Ai14^wt/mut^) mice. P2Y12-CreER-GFP mice were crossed with DTA mice to obtain P2Y12-CreER-GFP::DTA (P2Y12^+/CreER-GFP^::DTA^wt/mut^) mice. CX3CR1^GFP/GFP^ mice were crossed with C57BL/6 J mice to obtain CX3CR1^+/GFP^ mice. P2Y12-CreER-GFP (P2Y12^CreER-GFP/CreER-GFP^) mice were crossed with C57BL/6 J mice to obtain P2Y12^+/CreER-GFP^ mice. Mice of both sexes were utilized for the experiments unless specified. All the animals were housed in the specific pathogen-free (SPF) facility at the Animal Facility of Shenzhen Institute of Advanced Technology at Chinese Academy of Sciences, Department of Laboratory Animal Science at Fudan University and Animal Facility of Shanghai Mental Health Center at Shanghai Jiao Tong University School of Medicine. The animals were exposed to a 12-hour light/12-hour dark cycle and provided food and water *ad libitum*. The ambient temperature was kept at 20 °C to 26 °C, and the humidity was maintained between 40 and 70%.

### Chemicals and reagents

The chemical reagents were purchased from Sigma‒Aldrich, and the cell culture media were purchased from Invitrogen unless otherwise specified. The CSF1R inhibitor PLX5622 was purchased from SYSE Bio (Cat#: JP-2112). PLX5622 was formulated into the AIN-76A diet at 1.2 g of PLX5622 per kilogram of diet by SYSE Bio (Cat#: D20010801). The normal AIN-76A diet (control diet, CD) was purchased from SYSE Bio (Cat#: PD1001). Ovomucoid (Cat#: A003085) and penicillin/streptomycin/amphotericin B solution (P/S/A, Cat#: B540733) were procured from Sangon Biotech. Percoll (Cat#: 17-0891-02) was purchased from GE Healthcare. FcR blocking reagent (Cat#: 130-097-679) and conjugated monoclonal anti-ACSA-2 antibodies (Anti-ACSA-2 MicroBeads) (Cat#: 130-097-678) were obtained from Miltenyi Biotec. BODIPY™ 505/515 (BODIPY, Cat#: D3921) and pHrodo™ Red (Cat#: P36600) were purchased from Thermo Fisher Scientific. Recombinant complement C4b (Cat#: RPB305Mu01) was purchased from Cloud-Clone. GFAP ELISA kit (Cat#: ab233621) was procured from Abcam. Normal donkey serum and normal goat serum were purchased from Jackson ImmunoResearch. Hydrocortisone (NSC 10483, Cortisol) was purchased from Selleck (Cat#: S1696). Putrescine was obtained from Merck (Cat#: 51799). Insulin (human) was acquired from Selleck (Cat#: S6955). Recombinant Human FGF-basic (154 a.a.) was purchased from PeproTech (Cat#: 100-18B). Recombinant murine EGF was purchased from PeproTech (Cat#: 315-09). Prostaglandin (PG) F2α was procured from Yeasen (Cat#: 60811ES03). Recombinant complement 4b (C4b) (endotoxin removal) (Cat#: RPB305Mu01), complement component 4a (C4a) (endotoxin removal) (Cat#: RPA389Mu01), complement component 1, Q subcomponent A (C1qA) protein (endotoxin removal) (Cat#: RPD207Mu01) and complement component 3 (C3) protein (endotoxin removal) (Cat#: RPA861Mu01) were purchased from Cloud-Clone. Recombinant mouse complement component C2 protein CF (C2) was acquired from R&D Systems (Cat#: 6725-SE-010).

### Drug administration

To pharmacologically eliminate brain microglia, the mice were administered a PLX5622-formulated AIN-76A diet *at libitum* as previously described^27,31,35,36,75–77^. In contrast, the control mice were fed the normal AIN-76A diet (control diet, CD) *ad libitum*^27,31,35,36,75–77^. To sparsely label microglia, a low dose of tamoxifen (Sigma, C8267) at the dose of 15 mg per kg of body weight for CX3CR1-CreER::Ai14 or 50 mg per kg of body weight for P2Y12-CreER-GFP::Ai14 dissolved in corn oil (Aladdin, C116025) was intraperitoneally administered once to each mouse.

### *Rubcn* siRNA knockdown

siRNA oligonucleotides targeting mouse *Rubcn* (CTCAGAGTAACAGGACCTT) were synthesized by OBiO Technology (Shanghai). Astrocytes were transfected with 50 nM *Rubcn* siRNA or the scramble control using jetPRIME® in vitro DNA & siRNA transfection reagent (Polyplus, Cat#: 114-15) according to the manufacturer’s protocol. Seventy hours after transfection, astrocytes were collected for Western blot detection and functional assays.

### AAV transduction of mCherry and *Rubcn* shRNA

To specifically target astrocytes, we used AAV.PHP.eB expression plasmids^39^ expressing the mCherry reporter under the control of a truncated *Gfap* promoter (GfaABC1D). The short *Gfap* promoter is followed by a woodchuck hepatitis virus posttranscriptional regulatory element (WPRE) and growth hormone polyadenylation signal (pA). The plasmids are flanked by AAV inverted terminal repeats.

All AAV expression vectors were constructed, packaged and molecularly verified by OBiO Technology (Shanghai). The miR30-based shRNA AAV vector targeting mouse *Rubcn* (CTCAGAGTAACAGGACCTT) was used for in vivo *Rubcn* knockdown, as verified in our siRNA knockdown assay. The designed AAV plasmids for *Rubcn* shRNA and scramble were constructed as pAAV-GfaABC1D-mCherry-mir30 *Rubcn* shRNA-WPRE and pAAV-GfaABC1D-mCherry-scramble-WPRE, respectively.

The AAV vectors were intravenously injected into the mouse tail using ultrafine needle insulin syringes (BD). The 2-month-old C57BL/6 J, CX3CR1^+/GFP^ and RUBICON^−/−^ mice received a single intravenous injection of AAV PHP.eB *Gfap*-mCherry at 2 × 10^11^ vg/kg. AAV PHP.eB *Gfap*-mCherry-*Rubcn* shRNA and *Gfap*-mCherry-scramble control were intravenously injected into 2-month-old C57BL/6 J mice. These mice were tested for functional performance and sacrificed after 32 days for sample collection.

### Tissue preparation for histological experiments

The mice were deeply anesthetized with a mixture of ketamine hydrochloride (100 mg per kg of body weight) and xylazine (10 mg per kg of body weight) by intraperitoneal injection. The animals were then sequentially perfused with 0.9% saline and 4% paraformaldehyde (PFA) (Sigma, 441244) in 0.01 M PBS. The brains were carefully collected and then post-fixed in 4% PFA in 0.01 M PBS at 4 °C overnight.

### Cryosection preparation

The brains were dehydrated in 30% sucrose in 0.01 M PBS at 4 °C for 2–3 days. After being embedded in optimal cutting temperature compound (OCT, Tissue-Tek), the brains were frozen in liquid nitrogen and stored at −80 °C before sectioning. Tissues with the regions of interest were sectioned using a cryostat (Leica, CM1950) at a thickness of 30 μm.

### Immunofluorescent staining

The brain sections were first subjected to three 10~15-minute rinses with 0.01 M PBS. The samples were then blocked in 4% normal donkey serum (NDS, Jackson ImmunoResearch, 017-000-121) or normal goat serum (NGS, Jackson ImmunoResearch, 005-000-121) in 0.01 M PBS containing 0.3% Triton X-100 (Sigma‒Aldrich T8787) (PBST) at room temperature (RT) for 1 h. Subsequently, the samples were incubated with primary antibodies with 1% NDS or NGS in PBST at 4 °C overnight. After three rinses with PBST, the samples were reacted with fluorescent dye-conjugated secondary antibodies with 4’,6-diamidino-2-phenylindole (DAPI, 1:500, Sigma‒Aldrich, Cat#: D9542-10MG, Lot#: 118M4025V) in 1% NDS or NGS containing PBST at RT for 1.5 h. The samples were then well rinsed three times and carefully mounted with antifade mounting medium (Vectashield H-1000 or DAKO S3023).

The primary antibodies used in this study included the following: goat anti-mCherry (Biorbyt, Cat#: Orb11618, Lot#: J2446; 1:500), rat anti-CD31 (BD Biosciences, Cat#: 550274, Lot#: 9259767; 1:10), rabbit anti-IBA1 (Wako, Cat#: 019-19741 Lot#: WDK2121; 1:500), goat anti-IBA1 (Abcam, Cat#: ab5076, Lot#: GR3187278-2; 1:500), chicken anti-GFP (Abcam, Cat#: ab13970, Lot#: GR236651-12; 1:1,000), rabbit anti-S100β (Abcam, Cat#: ab52642, Lot#: GR252937-5; 1:300), rabbit anti-GFAP (Abcam, Cat#: ab7260, Lot#: GR297722-2; 1:300), rabbit anti-α-SMA (Abcam, Cat#: ab124964, Lot#: GR181740-66; 1:300), mouse anti-GFAP (Sigma‒Aldrich, Cat#: G3893, Lot#: 105M4784 V; 1:200), goat anti-PDGFR-β (R&D systems, Cat#: AF385, Lot#: BIWO619121; 1:200), rabbit anti-LC3 (Cell Signaling Technologies, Cat#: 4108, Lot#: 3; 1:100), rat anti-LAMP1 (1D4B) (Santa Cruz, Cat#: SC-19992, Lot#: C2715; 1:50), rabbit anti-PDGFR-α (Cell Signaling Technologies, Cat#: 3174 s, Lot#: 7; 1:500), mouse anti-CC1 (Millipore, Cat#: 14-0661-82, Lot#: 3129980; 1:200), rat anti-mouse C4 (Abcam, Cat#: ab11863, Lot#: GR3315169-2; 1:100), goat anti-GFP (Abcam, Cat#: AB6673, Lot#: GR3373716-2; 1:1,000), chicken anti-mCherry (Abcam, Cat#: ab205402, Lot#: GR3271744-8; 1:500), chicken anti-NESTIN (Abcam, Cat#: ab134017, Lot#: GR3291127-1; 1:200), rabbit anti-RFP (Abcam, Cat#: ab62341, Lot#: GR3319727-1; 1:1,000), rabbit anti-NeuN (Abcam, Cat#: ab177487, Lot#: GR3275122-6; 1:500), rabbit anti-MBP (Abcam, Cat#: ab40390, Lot#: GR297609-1; 1:200), rabbit anti-LAMININ (Sigma, Cat#: L9393-100UL, Lot#: 087M4889 V; 1:250), rabbit anti-GFP (Invitrogen, Cat#: A-11122, Lot#: 2273763; 1:1000), goat anti-OLIG2 (R&D, Cat#: AF2418, Lot#: UPA0719061; 1:400) and rabbit anti-PDGFα (Cell Signaling, Cat#: 3164 S, Lot#: 02/2020-6; 1:500).

Secondary antibodies conjugated to Alexa Fluor 488 (AF488), AF568 and AF647 were diluted 1:600 unless otherwise specified. These antibodies included the following: AF488 donkey anti-rabbit (Thermo Fisher, Cat#: A11008, Lot#: 1829924), AF488 donkey anti-rabbit (Jackson ImmunoResearch, Cat#: 711-545-152, Lot#: 146247), AF488 donkey anti-goat (Jackson ImmunoResearch, Cat#: 705-545-003, Lot#: 145270, 1:2,000), AF488 donkey anti-goat (Thermo Fisher, Cat#: A11055, 1687906), AF488 donkey anti-chicken (Jackson ImmunoResearch, Cat#: 703-545-155, Lot#: 126602 and 122188), AF568 donkey anti-rabbit (Thermo Fisher, Cat#: A10042, Lot#: 1964370), AF568 donkey anti-goat (Thermo Fisher, Cat#: A11057, Lot#: 1871957 and 1640316), AF568 donkey anti-rat (Abcam, Cat#: Ab175475, Lot#: GR142910-1), AF488 goat anti-rat (Abcam, Cat#: Ab150157, Lot#: GR3189353-1) and AF647 donkey anti-rat (Jackson ImmunoResearch, Cat#: 712-605-153, Lot#: 142891).

The validation data of each antibody are listed on the websites of the corresponding manufacturers.

### Primary cell cultures

Primary microglial and astrocyte cultures were established from the brains of P1 ~ P3 neonatal C57BL/6 J mice. After careful removal of the meninges and blood vessels, the mouse cortex was minced using a surgical blade and dissociated by gentle mechanical disruption in 0.025% trypsin for 5 min. The cell suspension was then quenched by 100% FBS and filtered through a 70-μm strainer. Primary microglia of high purity were isolated and purified from the mouse cortex as previously described with minimal modifications^44^. In brief, the cell/tissue-containing T75 flask was shaken at 180 rpm for 30 min on an orbital shaker. The microglia-containing supernatant was collected, spun down and cultured in Dulbecco’s modified Eagle’s medium (DMEM) supplemented with 10% FBS, 1% penicillin/streptomycin and 20% LADMAC-conditioned media (produced from the LADMAC cell line CRL-2420). The resulting cells were purified microglia and ready to use the next day. The purity of the primary microglia was systematically evaluated in our previous study^36,44^.

Primary astrocytes were isolated and purified from the mouse cortex as previously described^78,79^. In brief, the cells were centrifuged and resuspended in Dulbecco’s modified Eagle’s medium (DMEM) F-12 supplemented with 10% FBS and 1% penicillin/streptomycin (complete medium). The cells were then plated in a T75 flask, and the culture medium was changed every 3 to 4 days. Seven days later, microglia and OPCs were removed by shaking the flasks for 6 h at 240 rpm. The cells were then shaken for 18 h, and the culture medium was changed every 6 h. The purity of astrocytes was examined based on the expression of GFAP, S100β, IBA1, PDGFR-β, CD31, α-SMA, PDGFR-α, CC1, TUJ1 and NESTIN.

For the serum-free primary astrocyte culture, one day after the purified astrocytes were plated, the serum-containing medium was removed by gently washing astrocytes with PBS three times and washing them with serum-free medium. The astrocytes devoid of serum were then cultured in chemically defined medium (CDM, serum-free medium supplemented with defined chemicals and growth factors), which promotes astrocyte survival but not reactivity, as previously described with minor modifications^48,49^. The CDM contains DMEM F-12 supplemented with 1.2 mg/mL NaHCO_3_, 50 nM hydrocortisone, 100 nM putrescine, 500 ng/mL prostaglandin F2a, 50 ug/mL insulin, 100 ng/mL fibroblast growth factor (FGF), 200 ng/mL epidermal growth factor (EGF) and 1% penicillin/streptomycin.

### In vitro debris phagocytosis assay

Microglial cell death was induced by repeated freeze‒thaw cycles (3X) as previously described^26^. The dead microglia were labeled with 2 μg/mL pHrodo™ Red at 37 °C for 30 min and then washed 3-4 times with 0.01 M PBS^80^.

To test whether the cell debris can be engulfed by cultured cells (e.g., primary microglia and astrocytes), pHrodo-labeled debris was added to the cell cultures at a debris-to-cell ratio of 10:1. The non-engulfed debris was thoroughly washed away with 0.01 M PBS. The engulfment of debris was then analyzed using a Nikon A1 laser-scanning confocal microscope.

To test the role of complement opsonization in the astrocytic phagocytosis of microglial debris in serum-containing culture medium, FBS was heated at 56 °C for 30 min to inactivate complement components as previously described^26^. The culture medium containing complement-inactivated FBS (complete medium-based formulation) was supplemented with 5 μg/mL C1q, C2, C3, C4a or C4b to test whether these supplements could restore debris engulfment in astrocytes.

To test the requirement of each complement component for the astrocytic phagocytosis of microglial debris in serum-free culture medium, we cultured astrocytes in CDM that lacked serum. The CDM was first supplemented with 2 μg/mL C1q, C2, C3, C4a or C4b. We then tested which complement-supplemented CDM could restore the astrocytic engulfment of microglial debris at 72 h. Alternatively, the cultured astrocytes were incubated with pre-opsonized microglial debris with 5 μg/mL C1q, C2, C3, C4a or C4b overnight at 4 °C, and the astrocytic phagocytosis of cell debris after 72 h of cell culture was then analyzed.

### Magnetic-activated cell sorting (MACS)

C57BL/6 J mice were administered the PLX5622-formulated AIN-76A diet or the normal AIN-76A diet. Two days after treatment, the mice were deeply anesthetized and perfused with cold 0.01 M PBS containing heparin. The mouse brains were then carefully harvested and minced on ice immediately. Subsequently, the minced brain was trypsinized in a shaker at 37 °C and 100 rpm for 20 min, followed by the ovomucoid (2 mg/mL) neutralization. Three milliliters of L15 culture medium containing 0.5% bovine serum albumin (BSA) was then added, and the mixture was pipetted up and down. The upper-layer cell suspension was then passed three times through a 100-μm cell strainer. The cell suspension was transferred into a 15-mL tube, spun down and resuspended in L15 culture medium containing 30% Percoll. The myelin debris in the upper layer was then removed by gradient centrifugation at 500 × *g* for 10 min. The cell pellets were resuspended and blocked in L15 medium containing 0.5% BSA and 10% FcR blocking reagent at 4 °C for 10 min. The resuspended cells were then incubated with 10% anti-ACSA-2 MicroBeads at 4 °C for 15 min. The cells were then rinsed and resuspended in L15 medium containing 0.5% BSA. Astrocytes were enriched by flowing the cell suspension through the LS column (Miltenyi, 130-042-401) attached to a QuadroMACS separator (Miltenyi, 130-091-051) and washing the column three times with 3 mL of L15 medium containing 0.5% BSA. Immediately, the column was removed from the separator, and the magnetically labeled cells were flushed out with 5 mL of L15 medium containing 0.5% BSA. To increase the purity of ACSA-2-positive cells, the magnetic separation process was repeated two more times. The purified ACSA-2-positive cells were translocated in TRIzol and rapidly frozen in liquid nitrogen for subsequent RNA sequencing and qPCR.

### Fluorescence-activated cell sorting (FACS)

For the RNA-seq analysis of astrocytes, FACS was utilized for the harvesting of tdTomato^+^ cells from tamoxifen-administered ALDH1L1-CreER::Ai14 mice. In brief, adult ALDH1L1-CreER::Ai14 mice were deeply anesthetized with a mixture of ketamine hydrochloride (100 mg per kg of body weight) and xylazine (10 mg per kg of body weight) by intraperitoneal injection. The animals were then perfused with cold 1X PBS, and the brains were then dissociated immediately and cut into 1-mm^3^ pieces by mouse stainless steel brain matrices (RWD). The tissue pieces were subsequently transferred into a C tube containing 3 mL of papain (8 U/mL, Sangon Biotec, S501621) digestion buffer. The C tube was then attached onto a gentleMACS Octo Dissociator (Miltenyi) with the 37C_ABDK program. At the end of the program, the C tube was detached and briefly centrifuged at room temperature. Subsequently, 10 mL of ice-cold DBPS containing 0.5% BSA was added, and the mixture was then pipetted up and down with a 1-mL pipette until large tissue clumps were detached. The dissociated cells were then filtered through a 70-μm cell strainer (Falcon) and centrifuged at 300 × *g* and 4 °C for 10 min, and the supernatant was discarded. The cells were resuspended in 4 mL of 30% Percoll (Sigma‒Aldrich) and centrifuged at 700 × *g* and 4 °C for 10 min to remove debris. The cells were washed once with 3 mL ice-cold DBPS containing 0.5% BSA, and the dissociated cells were collected for subsequent cell sorting. Before loading the cells onto a MoFlo Astrios EQ Cell Sorter (Beckman Coulter), the cells were stained with pSIVA-FITC (Abcam), which labels dead cells. After removal of the doublets and cell debris by FSC/SSC, approximately 1.2 to 1.4 × 10^5^ pSIVA-FITC^-^ (488, 530/30) tdTomato^+^ (561, 585/42) cells were sorted for subsequent analysis by pSVIA-FITC. The cell sorter was controlled by Summit, and the FACS data were analyzed using FlowJo 10.4.

### RNA sequencing

After astrocytes were harvested by FACS, total RNA was extracted using TRIzol. The RNA purity and quantification were evaluated with a NanoDrop 2000 instrument (Thermo). The RNA integrity was assessed with an Agilent 2100 instrument (Agilent Technologies). Libraries were then constructed using a TruSeq Stranded mRNA LT Sample Prep Kit (Illumina) according to the manufacturer’s instructions. Transcriptome sequencing and analysis were conducted by OE Biotech. The libraries were then sequenced by Illumina HiSeq X Ten with the 150 bp paired-end (150 PE) platform, and 43.50 M to 50.84 M raw reads were generated from each sample. The raw fastq data were processed by Trimmomatic^81^, low-quality reads were removed to obtain clean reads, and 41.41 M to 48.53 M clean reads were obtained from each sample. The clean reads were mapped to the mm10 mouse genome using HISAT2^82^. The FPKM of each gene was calculated with Cufflinks^83^. The read counts of each gene were then obtained by HTSeq-count^84^. Differential expression was analyzed using edgeR 3.28.1^85^. The DEG thresholds were set to |fold change | >= 2 and FDR <= 0.01.

### Quantitative PCR (qPCR)

Total RNA from cultured cells or MACS-sorted ACSA-2-positive cells was extracted with TRIzol. cDNA was reverse transcribed from total RNA using the ReverTra Ace™ qPCR RT kit (FSQ-101, TOYOBO) according to the manufacturer’s instructions. Subsequently, a 10-μL reaction system was prepared for qPCR using FastStart Essential DNA Green Master Mix (Roche, 06402712001) with a LightCycler 96 real-time PCR system (Roche, 05815916001). The relative cDNA concentrations of target genes were normalized to *Gapdh*. The primers used in this study were synthesized by BGI (Shenzhen, China) and included the following:

*Gapdh*-forward (TGAGGCCGGTGCTGAGTATG −3ʹ),

*Gapdh*-reverse (TGGTTCACACCCATCACAAACA),

*Gfap*-forward (CACCTACAGGAAATTGCTGGAGG),

*Gfap* -reverse (CCACGATGTTCCTCTTGAGGTG),

*C4b*-forward (GGAGAGTGGAACCTGTAGACAG),

*C4b*-reverse (CACTCGAACACGAGTTGGCTTG).

### Western blotting

The cultured astrocytes were lysed in cold RIPA buffer containing 1% protease inhibitor cocktail. The protein concentrations of the cleared lysates were determined using the bicinchoninic acid (BCA) assay. Twenty micrograms of total protein was loaded into and resolved by a 12% SDS‒PAGE gel and subsequently transferred to a 0.45-μm PVDF membrane using a wet transfer system (Bio-Rad). The membrane was blocked in 5% nonfat milk in TBST for 1 h and incubated with rabbit anti-RUBICON (D9F7) antibody (Cell Signaling Technologies, Cat#: 8465, Lot#: 2; 1:1,000) and mouse anti-ß-actin (Santa Cruz Biotechnology, Cat#: SC-47778, Lot#: A2317; 1:3,000) overnight at 4 °C. After three 5-minute TBST washes, the membranes were probed with goat anti-rabbit (HRP) (Dako, Cat#: P0448, Lot#: 20034870; 1:3,000). The washed membranes were developed with an ECL kit. The scanned images were processed using Adobe Photoshop CS4.

### Enzyme-linked immunosorbent assay (ELISA)

The cultured astrocytes were lysed in RIPA buffer with 1% protease inhibitor cocktail. The cell lysates were centrifuged at 19,000 g for 30 min at 4 °C. The protein concentration in the supernatants was measured using a BCA kit. The protein level of GFAP in cell lysates was analyzed against GFAP antibody with the GFAP ELISA kit (Abcam, Cat#: ab233621) according to the manufacturer’s instructions.

### BODIPY lipid staining

BODIPY staining was performed to detect intracellular neutral lipid accumulation in cultured astrocytes. The cell samples were fixed with 4% PFA in 0.01 M PBS for 20 min and rinsed three times with PBS. The fixed cells were then incubated with 2 μM BODIPY staining solution in 0.01 M PBS at 37 °C for 15 min and then washed three times with PBS. The stained samples were imaged with a laser-scanning confocal microscope.

### Confocal microscopy

Confocal images of fluorescent specimens were obtained with a Nikon A1 confocal microscope equipped with a 20 × 0.75 numeral aperture (NA) Plan Apo objective and a 60 × 1.49 NA oil immersion objective or a Carl Zeiss LSM 900 confocal microscope equipped with the laser module URGB (diode laser 405 nm; diode laser 488 nm; diode (SHG) laser 561 nm and diode laser 640 nm) and the Airyscan 2. Plan-Apochromat 20x (0.8 NA) objective. Confocal images were captured with a distance interval of 0.5 µm between z-sections. The *xy* view of the confocal images is presented as maximal projections of z stacks, and *xz* or *yz* slice views of the regions of interest were reconstructed to illustrate the protein colocalization. For cell culture, images were acquired by a single focal plane. All confocal images were captured and processed using Nikon NIS-Elements AR (v.4.6) or ZEN 3.0 (Carl Zeiss), whereas brightness, contrast and gamma correction were performed if necessary. Confocal images with Z-stacks were utilized for 3D reconstruction using Imaris 9.7 (Oxford Instruments) (debris engulfment) or ZEN 3.0 (Carl Zeiss) (whole-mount retina).

### Blood‒brain barrier integrity assay

The assay of the BBB integrity using dextran was mainly performed as previously described^86–89^ with modifications. In brief, mice were first fed a PLX5622-formulated AIN-76A diet or a control AIN-76A diet for 2 or 4 days. Subsequently, 75 μL of 10-kDa dextran-Alexa Fluor 647 (2 mg/mL) was injected through the mouse tail vein. Three hours after the injection, the mice were deeply anesthetized with a mixture of ketamine hydrochloride (100 mg per kg of body weight) and xylazine (10 mg per kg of body weight) via intraperitoneal injection. Subsequently, the mice were perfused with 0.9% saline to remove the residual tracer confined within blood vessels and then subjected to 4% PFA perfusion. The brain and liver were then collected and fixed with 4% PFA containing 0.01 M PBS at 4 °C overnight.

### Quantification analysis

The percentage of tdTomato-negative microglia engulfing tdTomato-positive microglia was quantified from at least 500 tdTomato-negative microglia (N = 5 mice for each group), as counted under the 40X objective through the eyepiece of a Nikon A1 confocal microscope. To carefully determine the presence of any tdTomato-positive microglia within tdTomato-negative microglia, the *z*-axis was adjusted back and forth to ensure that the fluorescent signals deep in the tissue were included. Representative images were captured at 60X.

The in vivo astrocyte phagocytosis of microglial debris was determined as the presence of GFP^+^ or IBA1^+^ puncta/fragments within mCherry-labeled astrocytes from approximately 700 to 1000 mCherry-labeled astrocytes in 5 to 7 mice. The majority of these counts were acquired from direct observation using a 40X objective from the eyepiece of a Nikon A1 confocal microscope. The *Z* axis was adjusted back and forth to ensure that all signals deep in tissue sections were included during the direct observation.

To quantify the phagocytosis of microglial debris by astrocytes in the retina and other cell types as stained with their markers or labeled by the transgene, we captured approximately 15 to 20 fields and analyzed the colocalization between microglial debris (CX3CR1-GFP or IBA1 staining) and other cell types (markers or *Nes*-GFP) using Nikon NIS-Elements AR software. The colocalization was quantitively presented as the Manders overlap coefficient (r). Similar quantification processes were applied to the astrocyte phagocytosis of microglial debris in the 5xFAD model (*N* = 5 mice) and C4 colocalization with microglial debris (*N* = 5 mice for in vivo experiments and *N* = 5 independent biological replicates for in vitro experiments).

The in vitro astrocyte phagocytosis of microglial debris was determined by quantifying the mean fluorescent intensity (MFI) of pHrodo-labeled microglial debris within astrocytes from several independent ×20 imaging fields (*N* = 5 independent biological replicates). The MFI was calculated using ImageJ (v.1.53), and the fold change was normalized against the control.

The in vivo GFAP intensity following PLX5622 administration was determined by measuring the MFI of GFAP signals from 3 to 5 independent fields per mouse (*N* = 5 mice).

The size of LC3-positive puncta in astrocytes treated with microglial debris or starvation was measured from 81 puncta of 3 independent biological replicates using ImageJ. Note that only LC3 puncta that encircle microglial puncta were included in the microglial debris-treated group. The numbers of LAPosomes in astrocytes were counted from at least 7 TEM fields (*N* = 4 and 5 independent biological replicates for the starvation and microglial debris-treated groups, respectively). Only those puncta showing the single-membrane structure with a diameter larger than 1 μm were included as putatively LAPosomes. The MFI of BODIPY lipids was quantified from 9 to 10 independent fields (*N* = 3 independent biological replicates) using ImageJ.

### Statistics and reproducibility

The statistical analyses were performed using Prism 8.4.0 (GraphPad) or R 3.6.1 (R Foundation). The results are presented as mean ± standard deviation (SD) (bar plot) or median ± quartile (violin plot). A two-tailed independent t test was used to compare the differences between two groups, whereas one-way analysis of variance (ANOVA) with Holm‒Sidak’s multiple comparisons test (post hoc) was used for comparisons among multiple groups. Statistical significance was defined as p (or adjusted p) ≤ 0.05. The exclusion criteria for experimental data points were death or severe sickness of animals during the experimental period. No outliers were excluded in this study. The animals and cultured cells were stochastically grouped from each experimental treatment or treatment condition. The results from the animal studies and cell cultures were evaluated independently by two blinded experienced researchers. No statistical methods were used to predetermine the sample sizes, but our sample sizes are similar to those reported in our previous publications^26,27,31,35,36,90–92^. Each experiment was repeated in at least two independent batches to avoid bias among a single batch. Several biological replicates were included in each independent batch. A reanalysis of RNA-seq data (accession code GEO ID: GSE108269)^27^ was conducted using R (3.6.3) packages, including edgeR (3.28.1), pheatmap (1.0.12), cowplot (1.0.0), EnhancedVolcano (1.4.0), org.Mm.eg.db (3.10.0), clusterProfiler (3.14.3) and VennDiagram (1.6.20).

### Reporting summary

Further information on research design is available in the Nature Research Reporting Summary linked to this article.

## Supplementary information

Supplementary Information

Supplementary Data 1

Reporting Summary

## Data Availability

The data that support the findings of this study are available from the corresponding author Bo Peng at Fudan University within three months upon typical request. The RNA-seq raw data of the astrocytes generated in this study have been deposited in Gene Expression Omnibus under the accession code GSE171321. The raw DNA sequencing data for C4b^−/−^ mouse genotyping are available in Supplementary Data 1. Source data are provided with this paper.

## Source data

Source Data
