# Supplementary material for: Microglial debris is cleared by astrocytes via C4b-facilitated phagocytosis and degraded via RUBICON-dependent noncanonical autophagy in mice
Source: Nat Commun. 2022 Oct 24;13:6233. doi: 10.1038/s41467-022-33932-3 (PMC9592609; doi:10.1038/s41467-022-33932-3)
Supplement: Supplementary file 1 — Supplementary Information [file 41467_2022_33932_MOESM1_ESM.pdf]

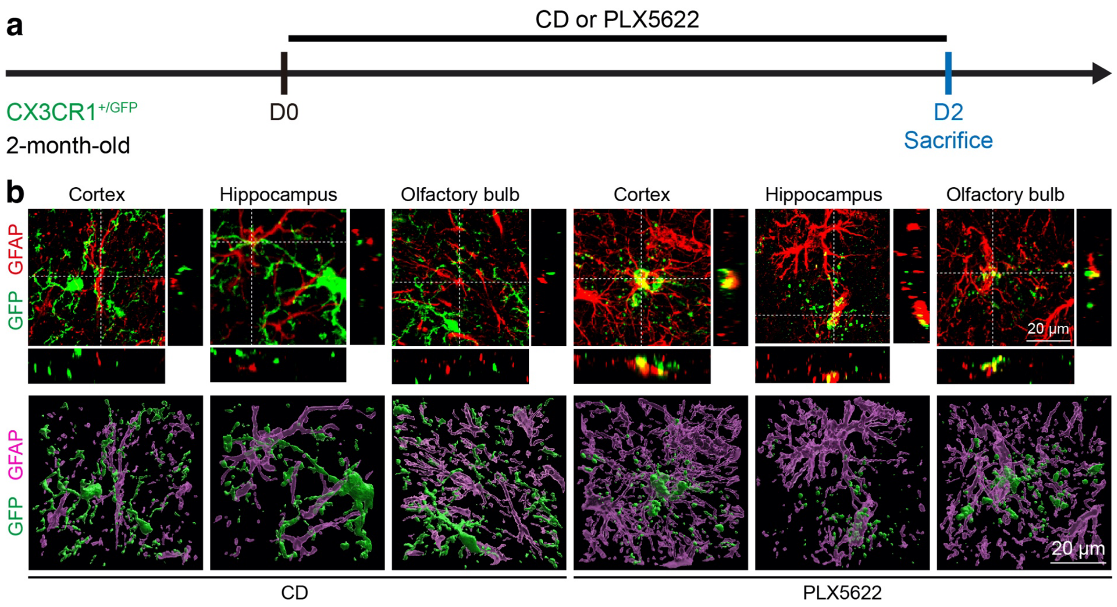

Supplementary Figure 1 GFAP staining reflects the astrocytic engulfment of microglial debris in the brain *in vivo*.

(a) Scheme of the astrocytic engulfment examination *in vivo* by GFAP-based astrocyte labeling and microglial depletion *in vivo*.

(b) Confocal orthogonal colocalization and 3D reconstruction show that GFAP^{+} astrocytes engulf GFP^{+} microglial debris upon the CSF1R inhibition *in vivo*. Each experiment is independently repeated from 6 mice with similar results.

PLX5622: PLX5622-formulated AIN-76A diet; CD: control AIN-76A diet.

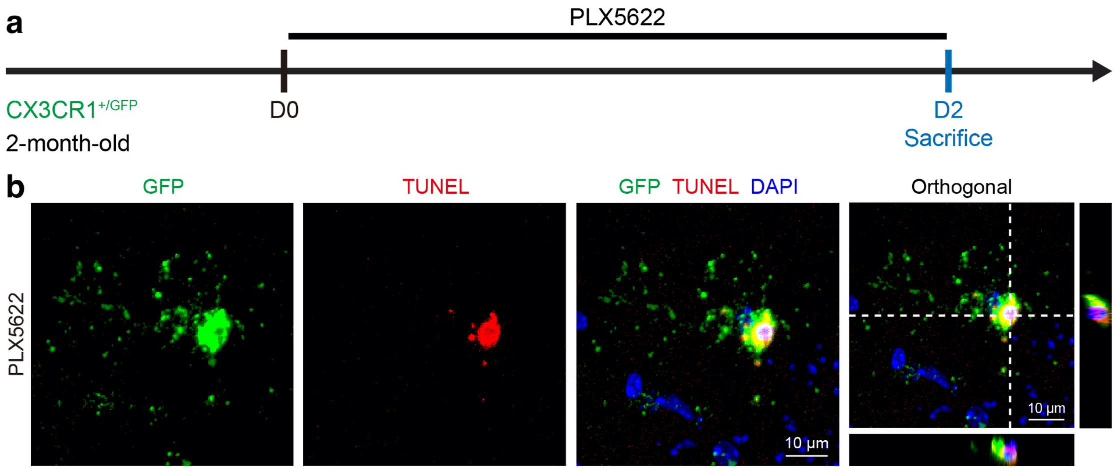

Supplementary Figure 2 Dying microglial cell is fragmented upon the CSF1R inhibition *in vivo*.

(a) Scheme of microglial depletion and time point for examination *in vivo*.

(b) Confocal images show that dying microglia are fragmented on pharmacological depletion *in vivo*. Each experiment is independently repeated from 4 mice with similar results.

PLX5622: PLX5622-formulated AIN-76A diet; CD: control AIN-76A diet.

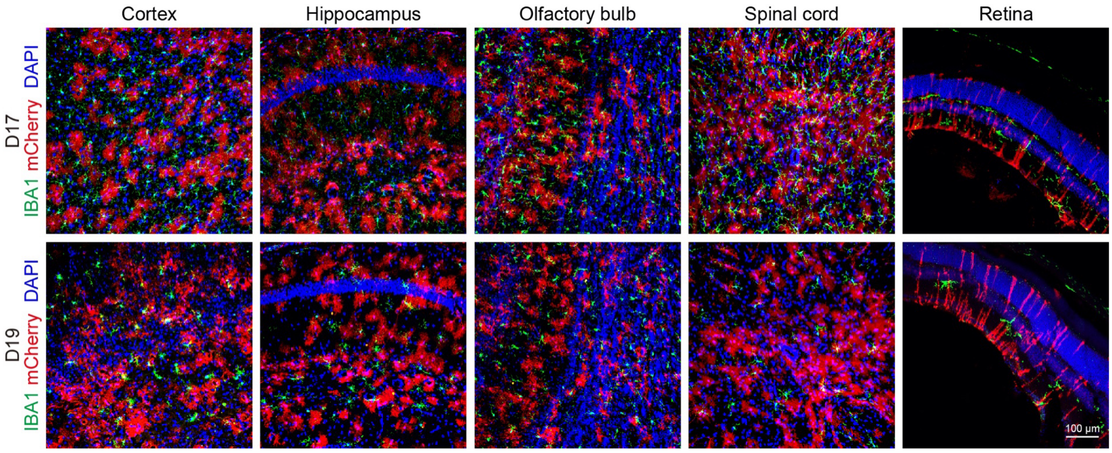

Supplementary Figure 3 Low power images of ALDL1L1-CreER::Ai14 astrocytes upon PLX5622 administration for 2 days *in vivo*. Experiments are independently repeated from 6 (D17) or 5 (D19) mice with similar results.

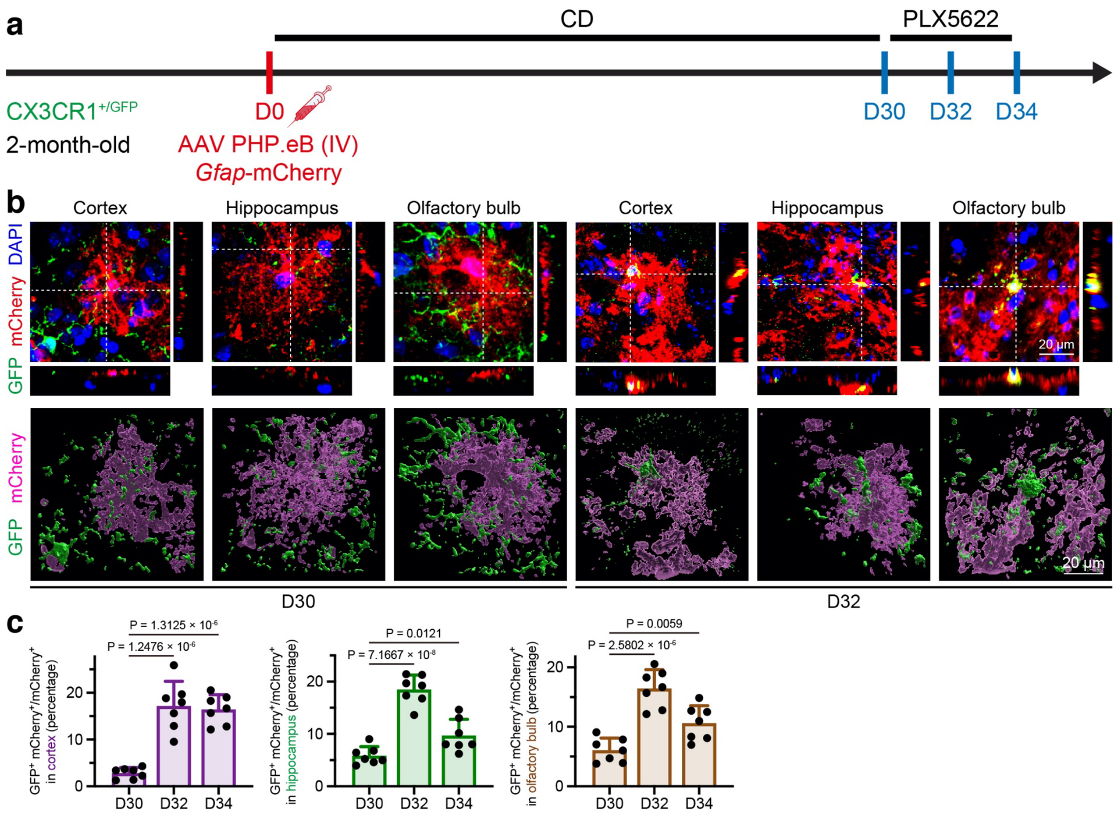

Supplementary Figure 4 Evidences from AAV PHP.eB demonstrate that brain astrocytes are capable of engulfing microglial debris *in vivo*.

(a) Scheme of the *in vivo* astrocytic engulfment examination by AAV PHP.eB-based astrocyte labeling and microglial depletion.

(b) Confocal orthogonal colocalization and 3D reconstruction show that mCherry$^+$ astrocytes do not engulf GFP$^+$ microglial debris at physiological condition (D30) whereas they engulf GFP$^+$ microglial debris upon the CSF1R inhibition (D32).

(c) Quantifications of the microglial debris engulfment by astrocytes. Cortex: 2.83% ± 1.18 (D30), 17.21% ± 5.24% (D32) and 16.44% ± 3.14% (D34); hippocampus: 5.86% ± 1.70% (D30), 18.47% ± 2.78% (D32) and 9.71% ± 3.08% (D34); olfactory bulb: 6.07% ± 2.04% (D30), 16.44% ± 3.14% (D32) and 10.63% ± 2.91 (D34). N = 7 mice for each group. One-way ANOVA with Holm-Sidak's multiple comparisons test (post hoc).

PLX5622: PLX5622-formulated AIN-76A diet; CD: control AIN-76A diet; IV: intravenous; MFI: mean fluorescence intensity. Data are presented as mean ± SD.

Source data are provided as a Source Data file.

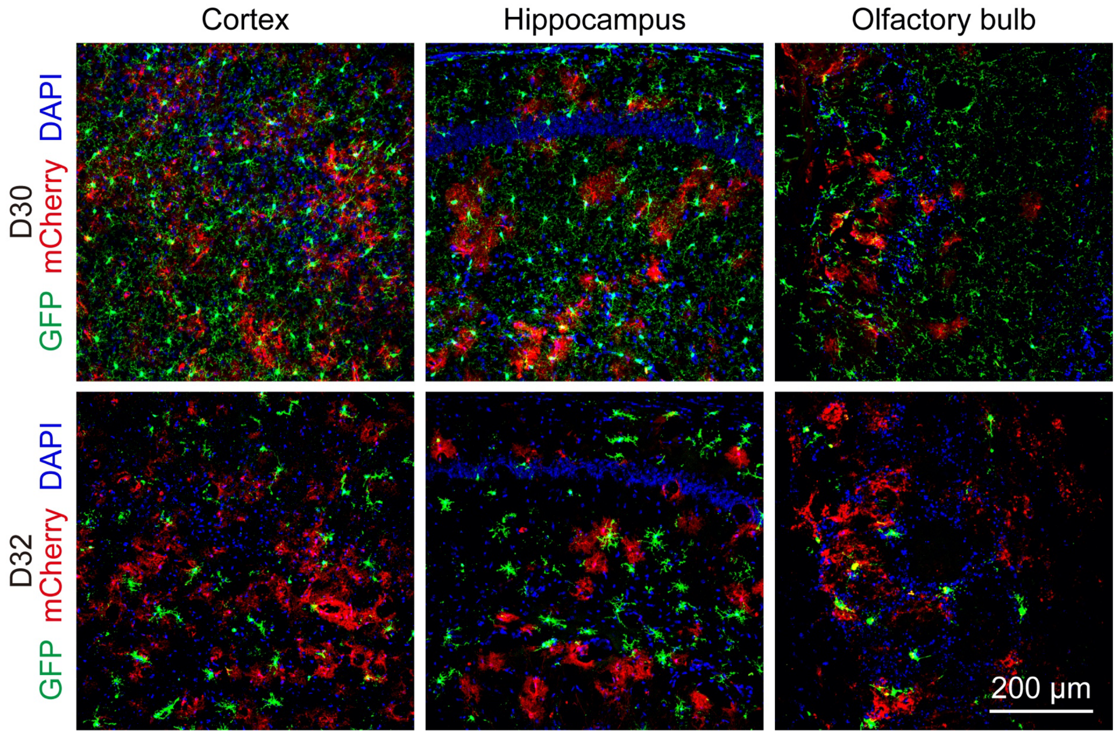

Supplementary Figure 5 Low power images of AAV PHP.eB *Gfap*-mCherry-labeled brain astrocytes upon PLX5622 administration for 2 days *in vivo*. Each experiment is independently repeated from 7 mice with similar results.

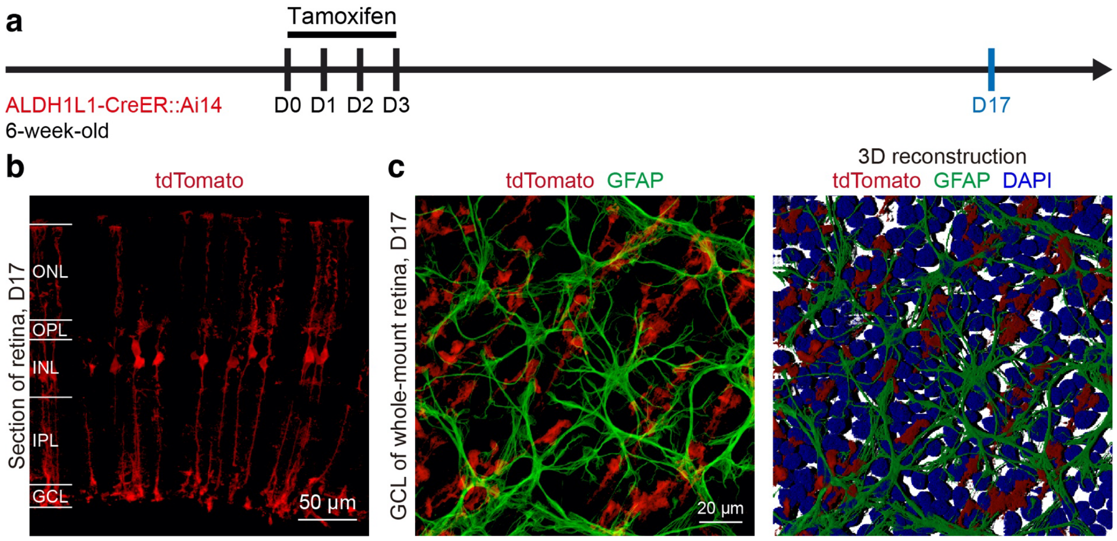

Supplementary Figure 6 Müller glia but not retinal astrocytes are specifically labeled in ALDH1L1-CreER::Ai14 mice *in vivo*.

(a) Scheme of the tamoxifen-induced tdTomato labeling in ALDH1L1-CreER::Ai14 mice.

(b) tdTomato expression in Müller glia are confirmed by the radial morphology and their INL-harbored cell bodies. Each experiment is independently repeated from 4 mice with similar results.

(c) Confocal images and 3D reconstruction show that retinal astrocytes in the GCL are not labeled by tdTomato. Each experiment is independently repeated from 4 mice with similar results.

ONL: outer nuclear layer; OPL: outer plexiform layer; INL: inner nuclear layer; IPL: inner plexiform layer; GCL: ganglion cell layer.

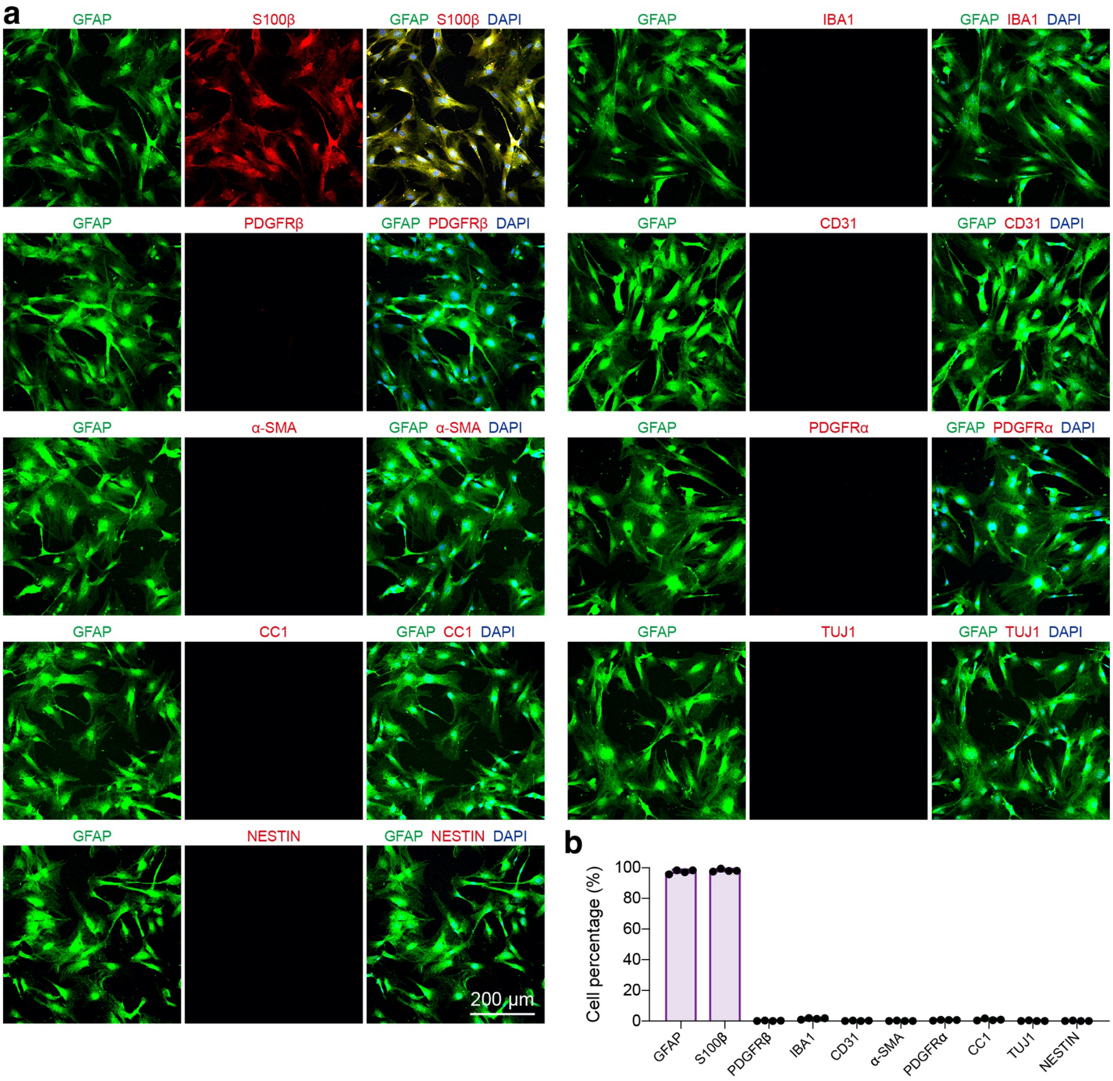

Supplementary Figure 7 Primary astrocyte cell culture of high purity *in vitro*.

(a) Almost all cells in the primary cell culture are astrocyte (GFAP and S100β), rather than microglia (IBA1), pericytes (PDGFR-β), endothelial cells (CD31), VSMCs (α-SMA), OPCs (PDGFR-α), oligodendrocytes (CC1), neurons (TUJ1) or neural stem cell (NESTIN).

(b) Quantification of astrocyte cell purity. N = 4 biological replicates for each group.

Data are presented as mean ± SD.

Source data are provided as a Source Data file.

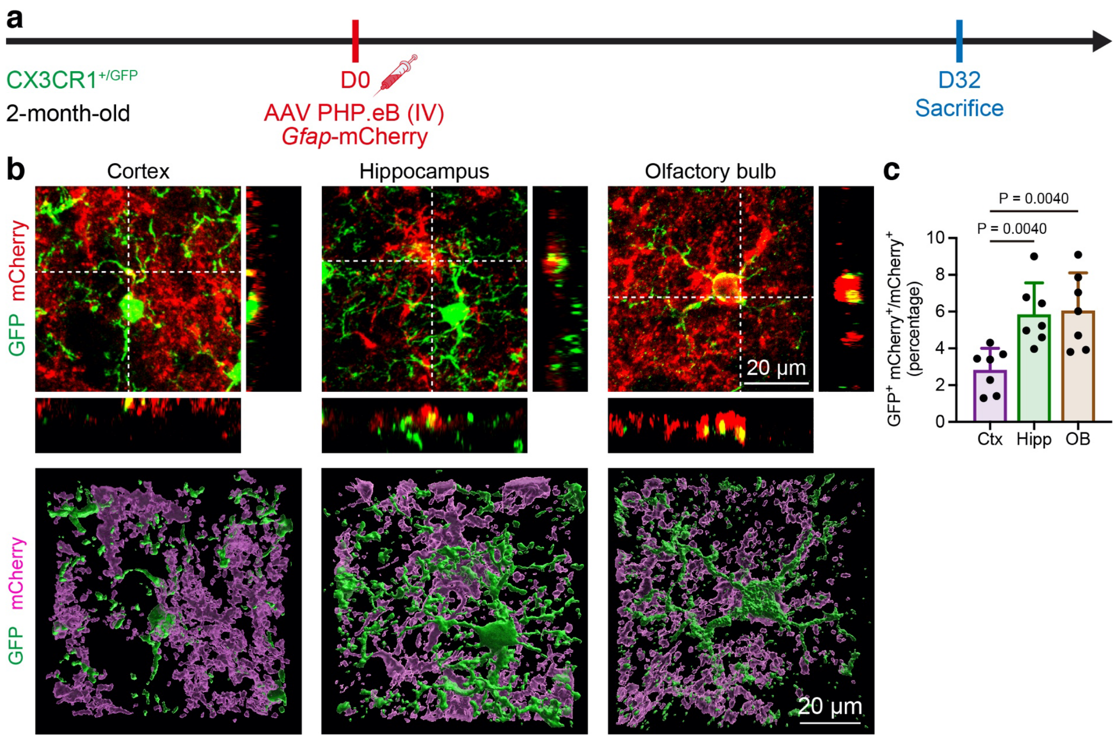

Supplementary Figure 8 AAV PHP.eB labeling confirms that the astrocytic engulfment of microglial debris is positively correlated with the speed of microglial turnover in the physiological condition *in vivo*.

(a) Scheme of the *in vivo* astrocytic engulfment examination in different brain regions at the physiological condition by AAV PHP.eB-based astrocyte labeling.

(b-c) Astrocytes in the cortex engulf less microglial debris than those in the hippocampus and olfactory bulb. 2.83% ± 1.18% (cortex), 5.86% ± 1.70% (hippocampus), and 6.07% ± 2.04% (olfactory bulb). N = 7 mice for each group. One-way ANOVA with Holm-Sidak's multiple comparisons test (post hoc). The quantitative results (c) are the re-analysis of same data from D30 group in Figure 1d.

Ctx: cortex; Hipp: hippocampus; OB: olfactory bulb; IV: intravenous. Data are presented as mean ± SD.

Source data are provided as a Source Data file.

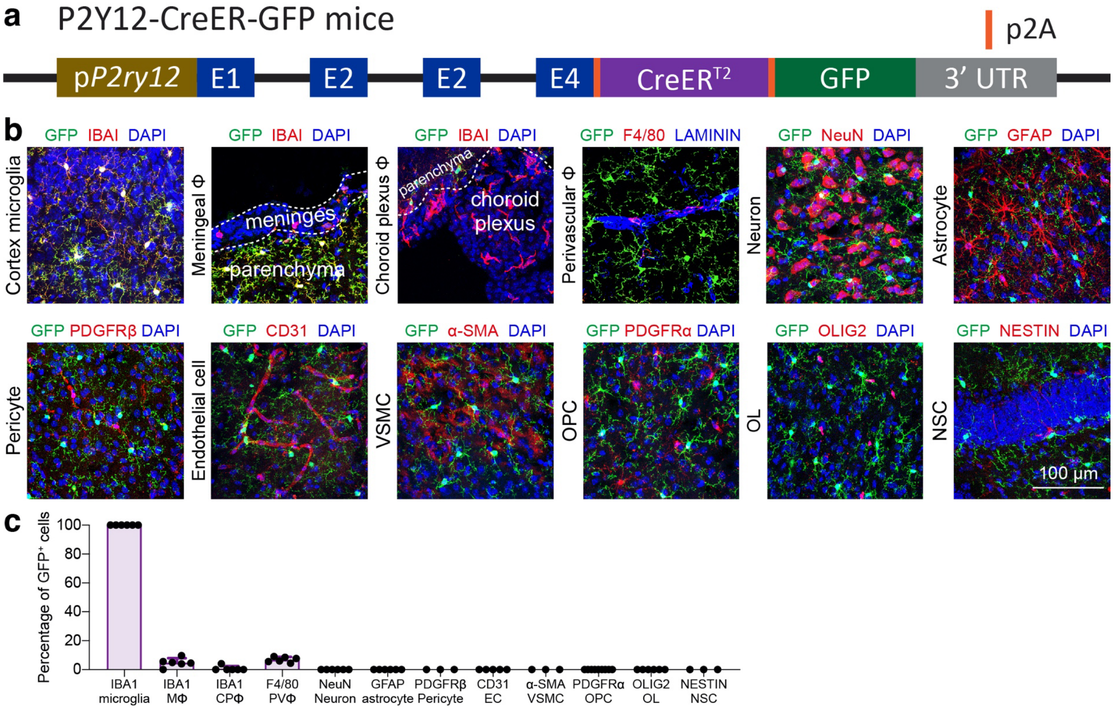

Supplementary Figure 9 P2Y12-CreER-GFP mouse specifically targets brain parenchymal microglia but not BAMs *in vivo*.

(a) Scheme of the targeting strategy for CreER-GFP knock-in at the *P2ry12* locus.

(b) Expression pattern of P2Y12-CreER-GFP in the mouse brain.

(c) Percentages of GFP^+ cells in the major cell types of the mouse brain. N = 6 mice for microglia, MΦ, CPΦ, PVΦ, neuron, astrocyte and OL, N = 3 for pericyte, VSMC and NSC, N = 5 for EC and N = 9 for OPC.

MΦ: meningeal macrophage; CPΦ: choroid plexus macrophage; PVΦ: perivascular macrophage; EC: endothelial cell; VSMC: vascular smooth muscle cell; OPC: oligodendrocyte precursor cell; OL: oligodendrocyte; NSC: neural stem cell. Data are presented as mean ± SD. Source data are provided as a Source Data file.

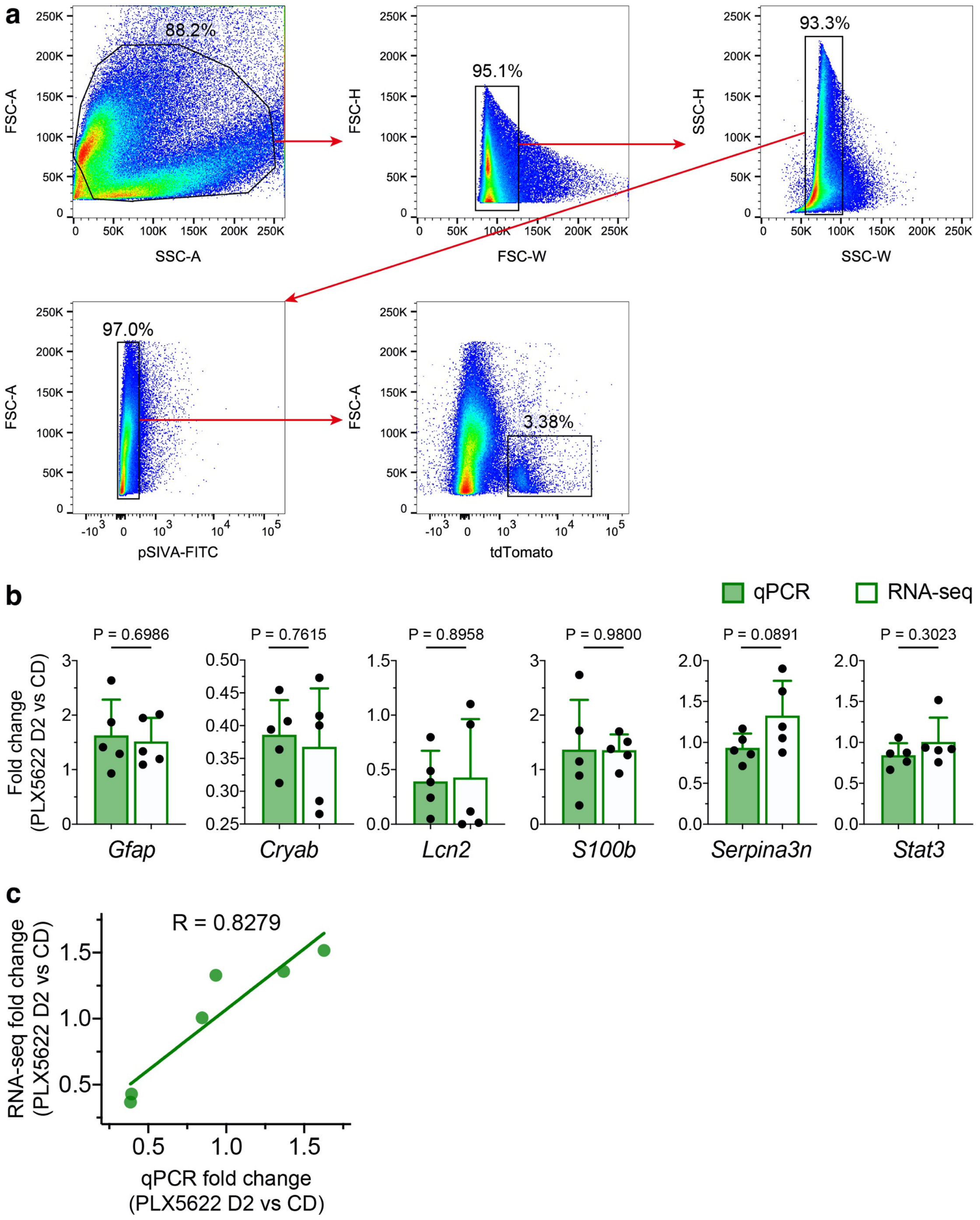

Supplementary Figure 10 Gating strategy for FACS and qPCR validation for the RNA-seq result *in vivo*.

(a) Gating strategy for the astrocyte sorting from the ALDH1L1-CreER::Ai14 mouse by FACS.

(b) Fold changes of a few reactive astrocyte marker genes by qPCR and RNA-seq (PLX5622 D2 vs CD).

Two-tailed independent t-test. N = 5 mice for each group.

(c) Linear regression shows that qPCR and RNA-seq results are positively correlated.

Samples are from independent experiments. Pearson correlation. N = 5 mice for each group.

Source data are provided as a Source Data file.

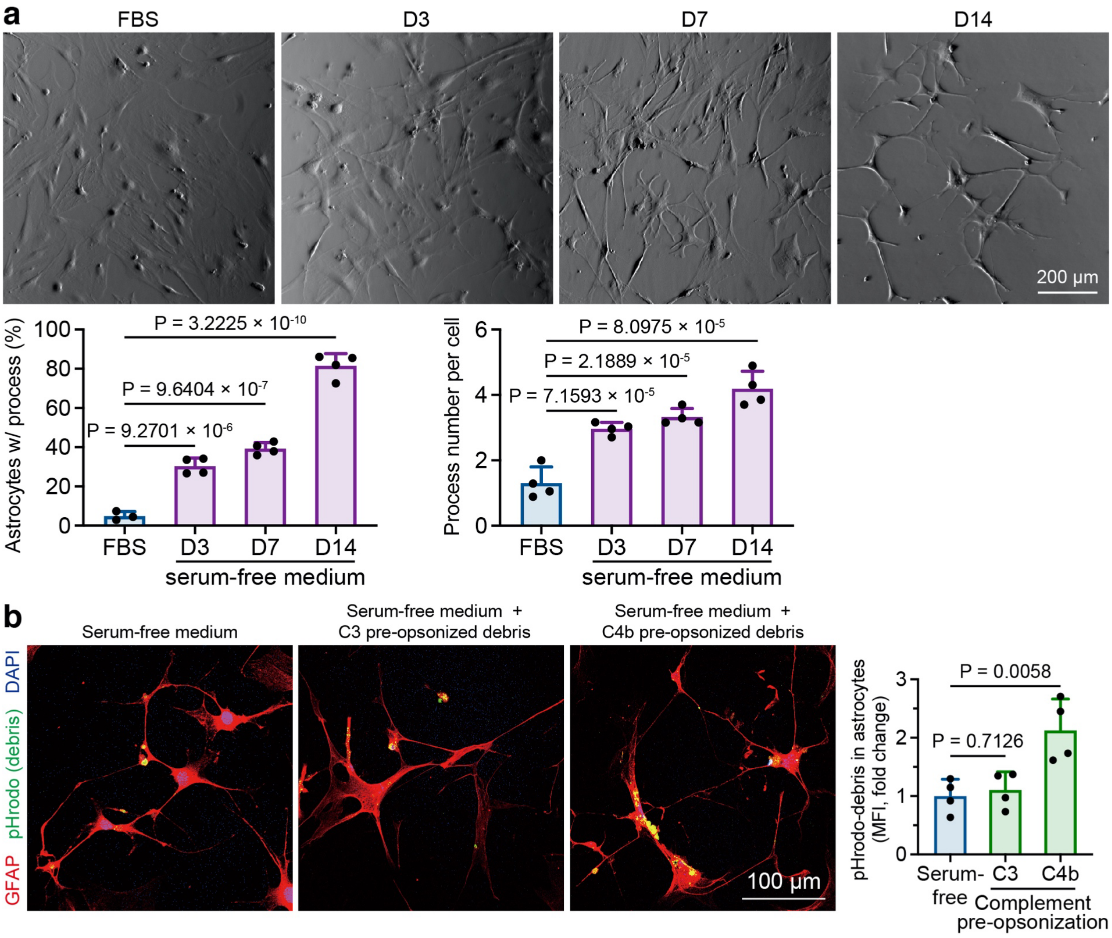

Supplementary Figure 11 C4b but not C3 facilitates the astrocytic engulfment of microglia debris in serum-free medium *in vitro*.

(a) Morphology of serum-free medium cultured astrocyte changes over time. N = 3 biological replicates for the PBS group the quantification of astrocytes without process, N = 4 biological replicates for the rest groups. One-way ANOVA with Holm-Sidak's multiple comparisons test (post hoc).

(b) C4b but not C3 facilitates the engulfment of microglia debris by process-bearing astrocytes cultured in serum-free medium. N = 4 independent biological replicates for each group. One-way ANOVA with Holm-Sidak's multiple comparisons test (post hoc).

Data are presented as mean ± SD.

Source data are provided as a Source Data file.

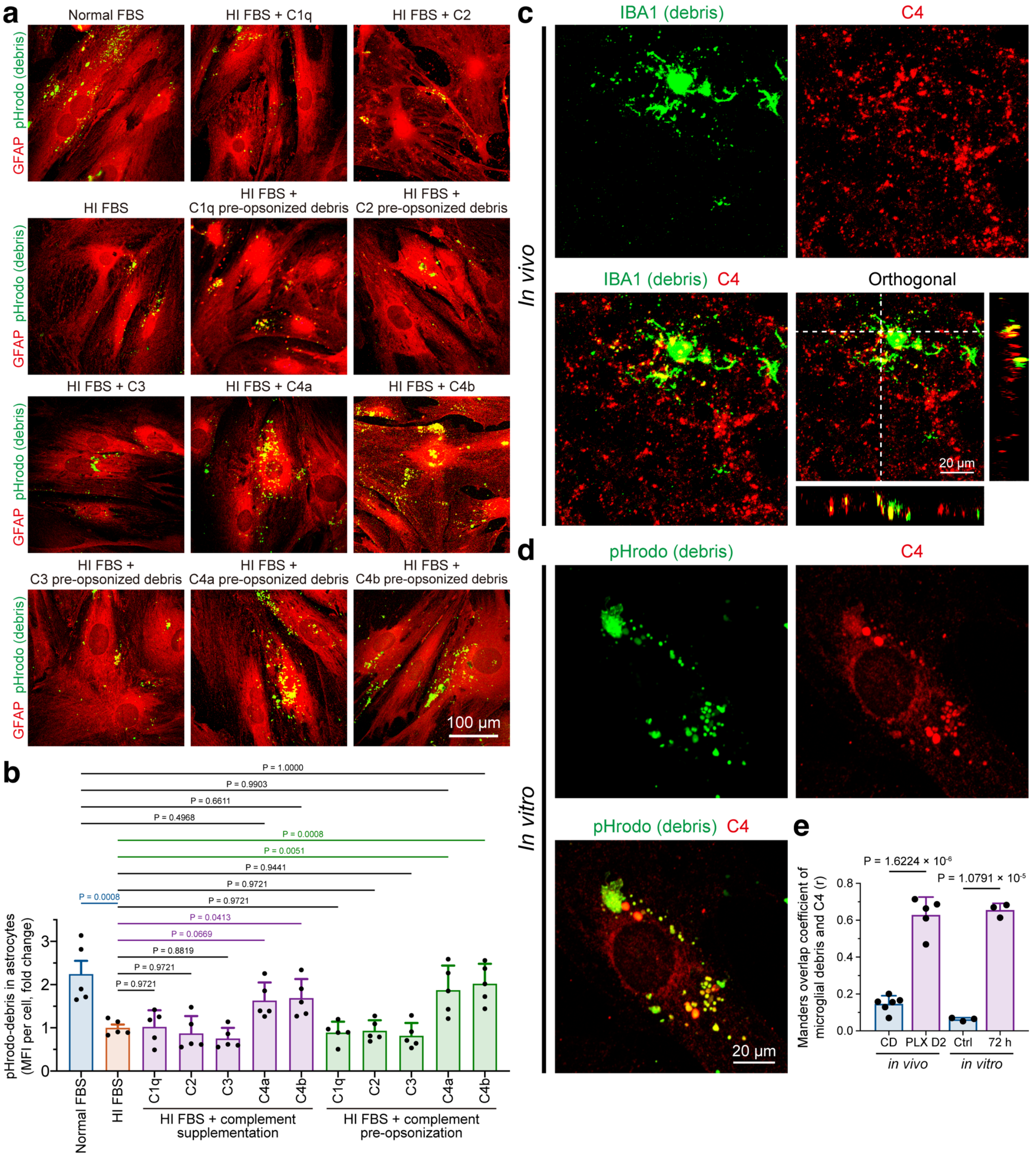

Supplementary Figure 12 C4a and C4b can facilitates the astrocytic engulfment of microglial debris *in vivo* and *in vitro*.

(a) Heat inactivation of complement components suppresses the microglial debris engulfment *in vitro*, whereas C4a and C4b restore the debris engulfment in the HI FBS-containing medium.

(b) Quantifications of the phagocytic influence by HI FBS, complement supplementation and pre-opsonization *in vitro*. N = 5 independent biological replicates for each group. One-way ANOVA with Holm-Sidak's multiple comparisons test (post hoc).

(c-d) Microglial debris is opsonized by C4 *in vivo* (ALDH1L1-CreER::Ai14 mice, 2 days after CD or PLX5622 administration) (c) and *in vitro* (72 hours after microglial debris and astrocyte co-culture) (d).

(e) Quantifications of C4-opsonized microglial debris *in vitro* and *in vivo*. N = 6 (CD) and 5 (PLX D2) mice *in vivo*, N = 3 biological replicates for each group *in vitro*. Two-tailed independent t-test.

HI FBS: heat inactivated FBS; MFI: mean fluorescence intensity; PLX: PLX5622-formulated AIN-76A diet; CD: control AIN-76A diet. Data are presented as mean ± SD.

Source data are provided as a Source Data file.

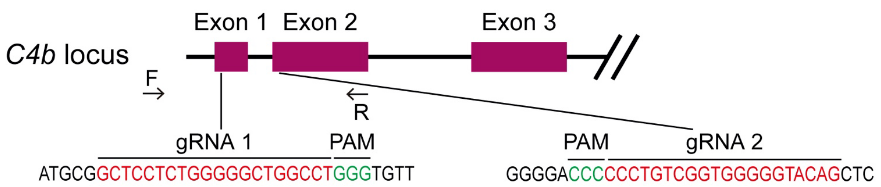

WT	TTCTGTGGTTAATTTGGGGACCCCCCTGTCGGTGGGGGTACAGCTCCTGGATGCCCCTCCAGGACAGGAGGT	
**F0** (biallelic)		Indel
# 4 homozygote	TTCTGTGGTTAATTTGGGGAC--------------------AGCTCCTGGATGCCCCTCCAGGACAGGAGGT	-20 bp
# 34 homozygote	TTCTGTGGTTAATTTGGGGACCCCCt--TCGGTGGGGGTACAGCTCCTGGATGCCCCTCCAGGACAGGAGGT	-3 bp, +1 bp
# 19 compound heterozygote	TTCTGTGGTTAATTT--------------CGGTGGGGGTACAGCTCCTGGATGCCCCTCCAGGACAGGAGGT	-14 bp
	TTCTGTGGTTAATTTGGGGACCCCCCT--CGGTGGGGGTACAGCTCCTGGATGCCCCTCCAGGACAGGAGGT	-2 bp
# 21 compound heterozygote	TTCTGTGGTTAATTTGGGGACCCCCCT-TCGGTGGGGGTACAGCTCCTGGATGCCCCTCCAGGACAGGAGGT	-1 bp
	TTCTGTGGTTAATTTGGGGACCCCCCTgtGTCGGTGGGGGTACAGCTCCTGGATGCCCCTCCAGGACAGGAGGT	+2 bp
# 40 compound heterozygote	TTCTGTGGTTAATTTGGG--------------TGGGGGTACAGCTCCTGGATGCCCCTCCAGGACAGGAGGT	-14 bp
	TTCTGTGGTTAATTT--------------CGGTGGGGGTACAGCTCCTGGATGCCCCTCCAGGACAGGAGGT	-14 bp
**F0** (multiallelic)		
# 6	TTCTGTGGTTAATTT -----------------TGGGGGTACAGCTCCTGGATGCCCCTCCAGGACAGGAGGT	-17 bp
	TTCTGTGGTTAATTTGGGG--------------GGGGGTACAGCTCCTGGATGCCCCTCCAGGACAGGAGGT	-14 bp
	TTCTGTGGTTAATT --------------TCGGTGGGGGTACAGCTCCTGGATGCCCCTCCAGGACAGGAGGT	-14 bp
	TTCTGTGGTTAATTT ------------- TCGGTGGGGGTACAGCTCCTGGATGCCCCTCCAGGACAGGAGGT	-13 bp
# 51	TTCTGTGGTTAATTTGGGG-------------------TACAGCTCCTGGATGCCCCTCCAGGACAGGAGGT	-19 bp
	TTCTGTGGTTAATTTGGG--------------TGGGGGTACAGCTCCTGGATGCCCCTCCAGGACAGGAGGT	-14 bp
	TTCTGTGGTTAATTTGGGGA-------------------ACAGCTCCTGGATGCCCCTCCAGGACAGGAGGT	-19 bp
	TTCTGTGGTTAATTTGGGG--------------GGGGGTACAGCTCCTGGATGCCCCTCCAGGACAGGAGGT	-14 bp
	TTCTGTGGTTAATTTGGGGACCCCC-------------------TCCTGGATGCCCCTCCAGGACAGGAGGT	-19 bp
# 54	TTCTGTGGTTAATTTGGGGACCCCCC--TCGGTGGGGGTACAGCTCCTGGATGCCCCTCCAGGACAGGAGGT	-2 bp
	TTCTGTGGTTAATTTGGGGACCCCCC----GGTGGGGGTACAGCTCCTGGATGCCCCTCCAGGACAGGAGGT	-4 bp
	TTCTGTGGTTAATTTGGGGACCCC----TCGGTGGGGGTACAGCTCCTGGATGCCCCTCCAGGACAGGAGGT	-4 bp
	TTCTGTGGTTAATTTGGGGAC--------------------AGCTCCTGGATGCCCCTCCAGGACAGGAGGT	-20 bp
	TTCTGTGGTTAATTTGGGGACCCC--------------------TCCTGGATGCCCCTCCAGGACAGGAGGT	-20 bp
	TTCTGTGGTTAATTTGGGGACC--------------------GCTCCTGGATGCCCCTCCAGGACAGGAGGT	-20 bp

Supplementary Figure 13 Indel analysis for CRISPR/Cas9 generated C4b$^{-/-}$ founder mutant mice (F0).

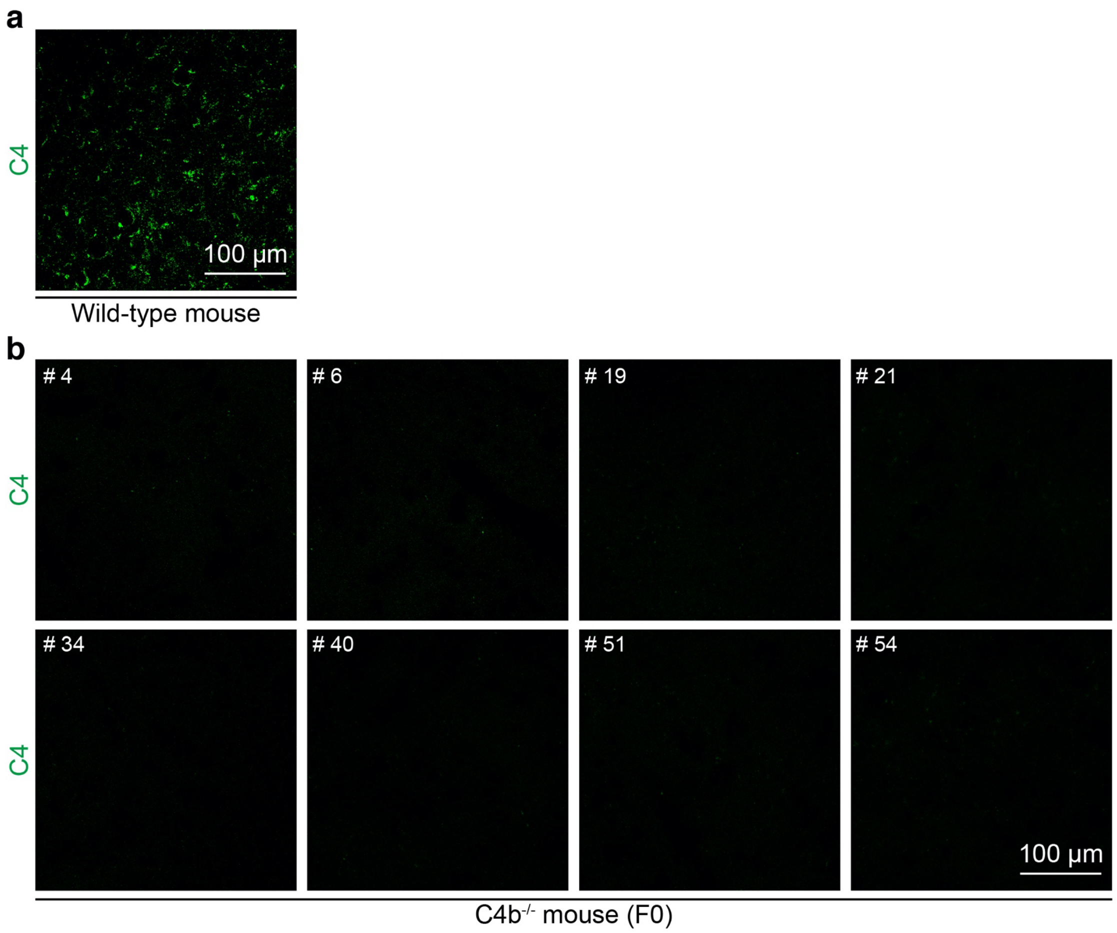

Supplementary Figure 14 Verification of C4 expression in C4b$^{-/-}$ founder mice (F0) *in vivo*.

(a) C4 expression in the brain of wild-type mouse. Each experiment is independently repeated from 4 mice with similar results.

(b) C4 expression in the brain of C4b$^{-/-}$ mouse (F0). Each experiment is independently repeated from 4 mice with similar results.

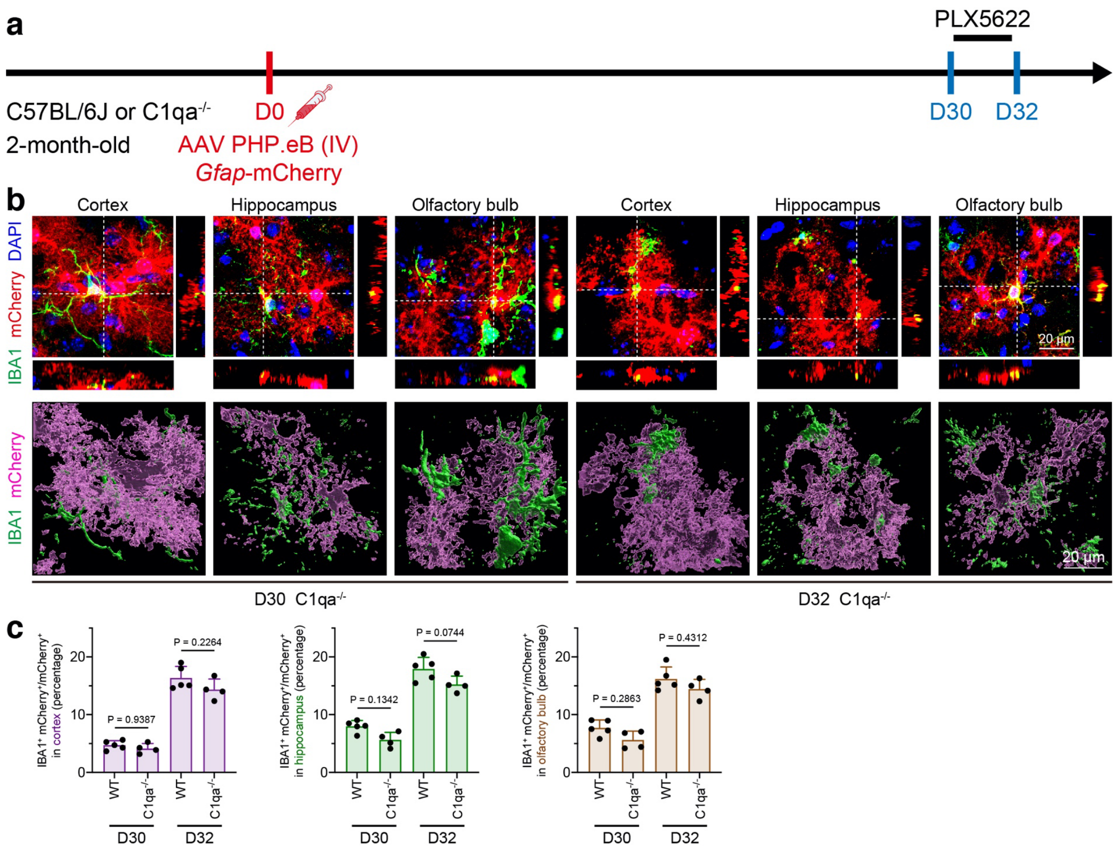

Supplementary Figure 15 C1q is not involved in the removal of microglial debris *in vivo*.

(a) Scheme of the *in vivo* examination of microglial debris removal in C1qa$^{-/-}$ mice by AAV PHP.eB-based astrocyte labeling and microglial depletion.

(b-c) C1qa knock-out does not influence the astrocytic engulfment of microglial debris in homeostasis (D30) or upon microglial ablation (D32). N = 5 (WT) and 4 (C1qa$^{-/-}$) mice for each group. One-way ANOVA with Tukey's multiple comparisons test (post hoc).

PLX5622: PLX5622-formulated AIN-76A diet; CD: control AIN-76A diet; IV: intravenous; WT: wild-type. Data are presented as mean ± SD.

Source data are provided as a Source Data file.

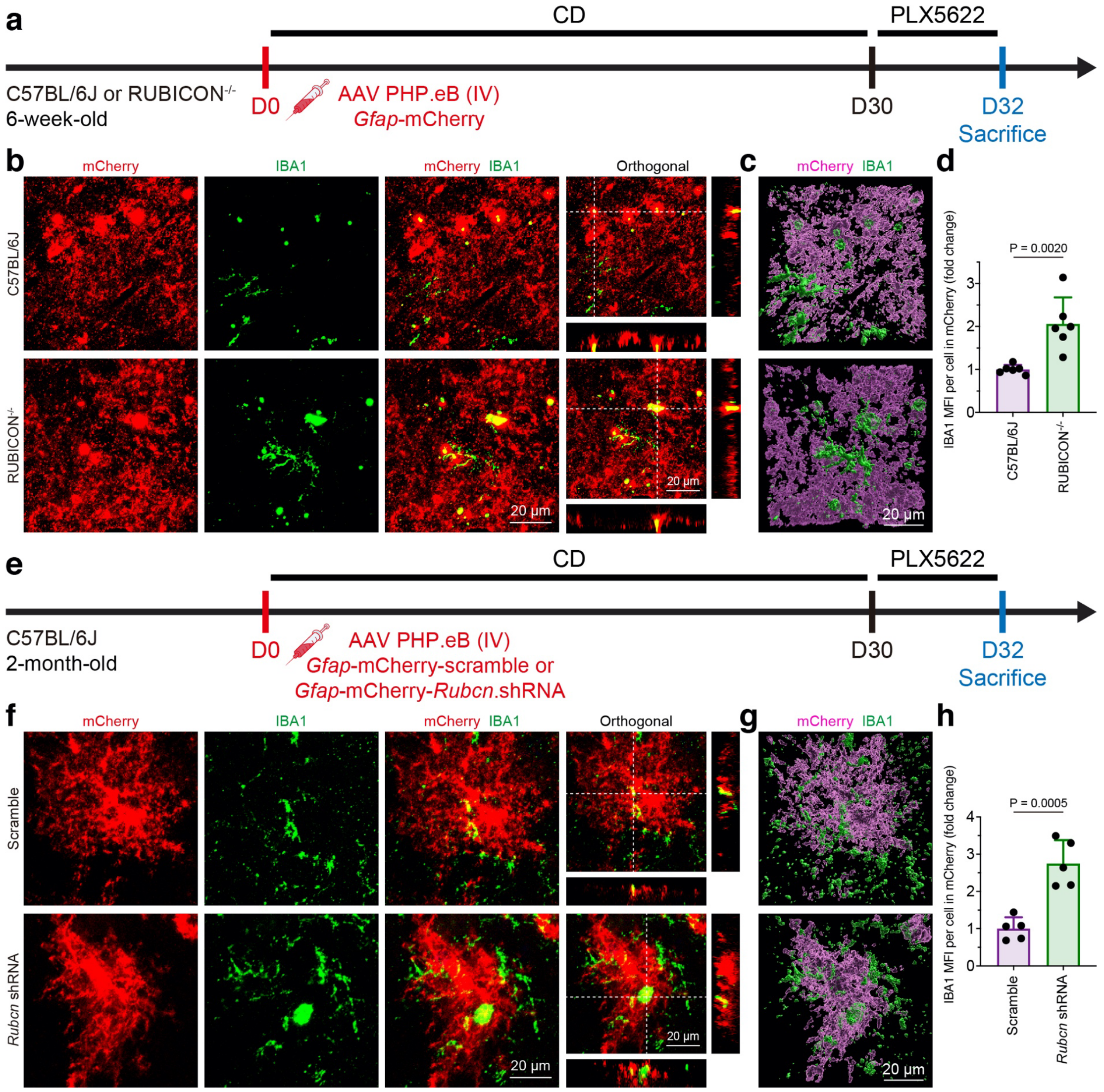

Supplementary Figure 16 Interruption of LAPosome formation by *Rubcn* knock-out or knock-down suppresses the degradation of engulfed microglial debris in astrocytes *in vivo*.

(a) Schemes of studying the *in vivo* consequence of total RUBICON knock-out to the degradation of engulfed microglial debris in astrocytes.

(b-d) RUBICON total knock-out *in vivo* dampens the degradation of engulfed microglial debris and thus results in the accumulation of non-degraded debris in astrocytes. N = 6 mice for each group. Two-tailed independent t-test.

(e) Schemes of studying the *in vivo* consequence of *Rubcn* knock-down to the degradation of engulfed microglial debris in astrocytes.

(f-h) *Rubcn* knock-down *in vivo* dampens the degradation of engulfed microglial debris and thus results in the accumulation of non-degraded debris in astrocytes. N = 5 mice for each group. Two-tailed independent t-test.

PLX5622: PLX5622-formulated AIN-76A diet; CD: control AIN-76A diet; IV: intravenous; MFI: mean fluorescence intensity. Data are presented as mean ± SD.

Source data are provided as a Source Data file.
