# Supplementary material for: Microglial debris is cleared by astrocytes via C4b-facilitated phagocytosis and degraded via RUBICON-dependent noncanonical autophagy in mice
Source: Nat Commun. 2022 Oct 24;13:6233. doi: 10.1038/s41467-022-33932-3 (PMC9592609; doi:10.1038/s41467-022-33932-3)
Supplement: Supplementary file 3 — Reporting Summary [file 41467_2022_33932_MOESM3_ESM.pdf]

nature portfolio

Corresponding author(s): Bo Peng

Last updated by author(s): Aug 25, 2022

Reporting Summary

Nature Portfolio wishes to improve the reproducibility of the work that we publish. This form provides structure for consistency and transparency in reporting. For further information on Nature Portfolio policies, see our Editorial Policies and the Editorial Policy Checklist.

Statistics

For all statistical analyses, confirm that the following items are present in the figure legend, table legend, main text, or Methods section.

n/a	Confirmed	
☐	☒	The exact sample size (n) for each experimental group/condition, given as a discrete number and unit of measurement
☐	☒	A statement on whether measurements were taken from distinct samples or whether the same sample was measured repeatedly
☐	☒	The statistical test(s) used AND whether they are one- or two-sided<br>*Only common tests should be described solely by name; describe more complex techniques in the Methods section.*
☒	☐	A description of all covariates tested
☒	☐	A description of any assumptions or corrections, such as tests of normality and adjustment for multiple comparisons
☐	☒	A full description of the statistical parameters including central tendency (e.g. means) or other basic estimates (e.g. regression coefficient) AND variation (e.g. standard deviation) or associated estimates of uncertainty (e.g. confidence intervals)
☐	☒	For null hypothesis testing, the test statistic (e.g. F, t, r) with confidence intervals, effect sizes, degrees of freedom and P value noted<br>*Give P values as exact values whenever suitable.*
☒	☐	For Bayesian analysis, information on the choice of priors and Markov chain Monte Carlo settings
☒	☐	For hierarchical and complex designs, identification of the appropriate level for tests and full reporting of outcomes
☐	☒	Estimates of effect sizes (e.g. Cohen's d, Pearson's r), indicating how they were calculated

Our web collection on statistics for biologists contains articles on many of the points above.

Software and code

Policy information about availability of computer code

Data collection

Fluorescence-activated cell sorting (FACS)

For RNA-seq analysis of astrocyte, FACS was utilized to harvest tdTomato+ cells from tamoxifen-administered ALDH1L1-CreER::Ai14 mice. In brief, adult ALDH1L1-CreER::Ai14 mice were deep anaesthetized with a mixture of ketamine hydrochloride (100 mg per kg of body weight) and xylazine (10 mg per kg of body weight) by intraperitoneal injection. After that, animals were perfused with cold 1X PBS. Brains were then dissociated immediately and cut into 1 mm3 pieces by mouse stainless steel brain matrices (RWD). Next, tissue pieces were transferred into a C tube containing 3 mL papin (8 U/mL, Sangon Biotec, S501621) digestion buffer. The C tube was then attached onto a gentleMACS Octo Dissociator (Miltenyi) with 37C_ABDK program. At the end of the program, detached the C tube and briefly centrifuged at room temperature. Then, added 10 mL ice cold DBPS containing 0.5% BSA and pipetted up and down with 1 mL pipette until large tissue clumps were detached. Then filtered dissociated cells through a 70 µm cell strainer (Faclon). Centrifuged the cells at 300 g for 10 minutes at 4 ºC and discarded the supernatant. Resuspended cells in 4 mL 30% Percoll (Sigma-Aldrich) and centrifuged 700 g for 10 min at 4 ºC to remove debris. Washed the cell with 3 mL ice cold DBPS containing 0.5% BSA once and collected the dissociated cells for the subsequent cell sorting. Before loading the cells onto MoFlo Astrios EQ Cell Sorter (Beckman Coulter), cells were stained with pSIVA-FITC (Abcam) which was used for labeling death cells. After removal the doublelets and cell debris by FSC/SSC, approximately 1.2 to 1.4 × 105 pSIVA-FITC- (488, 530/30) tdTomato+ (561, 585/42) cells were sorted for subsequent analysis with pSVIA-FITC. Cell sorter was controlled by Summit.

RNA-sequencing

After astrocytes were harvested by FACS, total RNA was then extracted by TRIzol. RNA purity and quantification were evaluated by NanoDrop 2000 (Thermo). RNA integrity was assessed by Agilent 2100 (Agilent Technologies). After that, libraries were constructed by TruSeq Stranded mRNA LT Sample Prep Kit (Illumina) according to the manufacturer's instruction. The transcriptome sequencing and analysis were conducted by OE Biotech. Next, libraries were sequenced by Illumina HiSeq X Ten by 150 bp paired-end (150 PE). 43.50 M to 50.84 M raw reads were generated for each sample.

Confocal microscopy
Confocal Images of fluorescent specimens were taken with Nikon A1 confocal microscope equipped with a 20x 0.75 numeral aperture (NA) Plan Apo objective and a 60x 1.49 NA oil immersion objective, or Carl Zeiss LSM 900 confocal microscope equipped with laser module URGB (diode laser 405 nm; diode laser 488 nm; diode (SHG) laser 561 nm and diode laser 640 nm) and Airyscan 2. The Plan-Apochromat 20x (0.8 NA) objective was utilized. Confocal images were captured with a distance interval of 0.5 µm between z-sections. The xy view of confocal images were presented as maximal projections of z stacks, and xz or yz slice views at regions of interest were reconstructed to illustrate the protein colocalization. For cell culture, images were acquired by single focal plan. All confocal images were captured and processed using Nikon NIS-Elements AR (v.4.6) or ZEN 3.0 (Carl Zeiss), whereas brightness, contrast and gamma correction were adjusted if necessary. Confocal images with Z-stacks were utilized for 3D reconstruction by Imaris 9.7 (Oxford Instruments) (debris engulfment) or ZEN 3.0 (Carl Zeiss) (whole-mount retina).

Data analysis

Fluorescence-activated cell sorting (FACS)
FACS data were analyzed by FlowJo 10.4.

RNA-sequencing
Raw fastq data were processed by Trimmomatic83 and low-quality reads were removed to obtain the clean reads. 41.41 M to 48.53 M clean reads were obtained for each sample. Clean reads were mapped to the mm10 mouse genome by HISAT284. FPKM of each gene was calculated by Cufflinks85. Read counts of each gene were then obtained by HTSeq-count86. Differential expression was analyzed via edgeR 3.28.187. The DEG threshold was set to |fold change| >= 2 and FDR <= 0.01.

Confocal microscopy
All confocal images were captured and processed using Nikon NIS-Elements AR (v.4.6) or ZEN 3.0 (Carl Zeiss), whereas brightness, contrast and gamma correction were adjusted if necessary. Confocal images with Z-stacks were utilized for 3D reconstruction by Imaris 9.7 (Oxford Instruments) (debris engulfment) or ZEN 3.0 (Carl Zeiss) (whole-mount retina).

Quantification analysis
The percentage of tdTomato negative microglia engulfing tdTomato positive microglia was quantified from at least 500 tdTomato negative microglia (N = 5 mice for each group), as counted under the 40X objective through the eyepiece of Nikon A1 confocal microscope. To carefully determine the presence of any tdTomato positive microglia within tdTomato negative microglia, z axis was adjusted back and forth to ensure the fluorescent signals deep in tissue were included. Representative images were captured at 60X.

In vivo astrocyte phagocytosis of microglial debris was determined as the presence of GFP+ or IBA1+ puncta/fragments within mCherry labeled astrocytes, from around 700 to 1,000 mCherry-labeled astrocytes of 5 to 7 mice. The majority of these counts were acquired from the direct observation using 40X objective from the eyepiece of Nikon A1 confocal microscope. Z axis was adjusted back and forth to ensure all signals deep in tissue sections were included during the direct observation.

To quantify the phagocytosis of microglia debris by astrocyte in the retina and other cell types as stained with their markers or labeled by transgene, we captured around 15 to 20 fields and analyzed the colocalization between microglia debris (CX3CR1-GFP or IBA1 staining) and other cell types (markers or Nes-GFP) using Nikon NIS-Elements AR software. Colocalization was quantitively presented as Manders overlap coefficient (r). Similar quantification processes applied to astrocyte phagocytosis of microglia debris in 5xFAD model (N = 5 mice), and C4 colocalization with microglia debris (N = 5 mice for in vivo experiments and N = 5 independent biological replicates for in vitro experiments).

In vitro astrocyte phagocytosis of microglia debris was determined by quantifying the mean fluorescent intensity (MFI) of pHrodo-labeled microglia debris within astrocytes from several independent 20X imaging fields (N = 5 independent biological replicates). The MFI was calculated in ImageJ (v.1.53) and fold change was normalized against control.

In vivo GFAP intensity following PLX5622 administration was determined by measuring the MFI of GFAP signals from 3 to 5 independent fields each mouse (N = 5 mice).

The size of LC3 positive puncta in astrocytes treated with microglial debris or starvation was measured from 81 puncta of 3 independent biological replicates by ImageJ. Note that only LC3 puncta that encircle microglia puncta were included in the microglial debris-treated group. Numbers of LAPosomes in astrocytes were counted from at least 7 TEM fields (N = 4 and 5 independent biological replicates for the starvation and microglial debris treated groups, respectively). Only those puncta showing the single-membrane structure with the diameter larger than 1 µm were included as putatively LAPosomes. The MFI of BODIPY lipids was quantified from 9 to 10 independent fields (N = 3 independent biological replicates) by ImageJ.

For manuscripts utilizing custom algorithms or software that are central to the research but not yet described in published literature, software must be made available to editors and reviewers. We strongly encourage code deposition in a community repository (e.g. GitHub). See the Nature Portfolio guidelines for submitting code & software for further information.

Data

Policy information about availability of data

All manuscripts must include a data availability statement. This statement should provide the following information, where applicable:

- Accession codes, unique identifiers, or web links for publicly available datasets
- A description of any restrictions on data availability
- For clinical datasets or third party data, please ensure that the statement adheres to our policy

The data that support the findings of this study are available from the corresponding author Bo Peng at Fudan University for reasonable request. The RNA-seq raw data of astrocytes generated in this study have been deposited in Gene Expression Omnibus under accession code GSE171321 [https://www.ncbi.nlm.nih.gov/geo/query/acc.cgi?acc=GSE171321]. The DNA sequencing raw data for C4b-/- mouse genotyping is available in Supplementary Data 1. Source data are provided with this paper.

Please note that, we did not deposit the DNA sequencing raw data for C4b-/- mouse in GEO, since these are genotyping data. GEO did not accept genotyping data. Therefore, we deposit this dataset in Supplementary Data 1.

Field-specific reporting

Please select the one below that is the best fit for your research. If you are not sure, read the appropriate sections before making your selection.

☒ Life sciences ☐ Behavioural & social sciences ☐ Ecological, evolutionary & environmental sciences

For a reference copy of the document with all sections, see nature.com/documents/nr-reporting-summary-flat.pdf

Life sciences study design

All studies must disclose on these points even when the disclosure is negative.

Sample size

For in vivo study (e.g. animal experiments), sample size was determined as the mouse number. For in vitro experiments, sample size was determined as the independent biological replicate. No statistical methods were used to predetermine the sample size, but our sample sizes are similar to those generally employed in the field. At least 3-5 mice were used in each group. This is consistent to our previous studies1-5 and others' glial studies6,7.

References

1. Niu, F. et al. The m6A reader YTHDF2 is a negative regulator for dendrite development and maintenance of retinal ganglion cells. Elife 11, e75827 (2022).
2. Rao, Y. et al. NeuroD1 induces microglial apoptosis and cannot induce microglia-to-neuron cross-lineage reprogramming. Neuron 109, 4094-4108.e5 (2021).
3. Xu, Z. et al. Efficient Strategies for Microglia Replacement in the Central Nervous System. Cell Rep 32, 108041 (2020).
4. Huang, Y. et al. Dual extra-retinal origins of microglia in the model of retinal microglia repopulation. Cell Discov 4, 9 (2018).
5. Huang, Y. et al. Repopulated microglia are solely derived from the proliferation of residual microglia after acute depletion. Nat Neurosci 21, 530-540 (2018).
6. Favuzzi, E. et al. GABA-receptive microglia selectively sculpt developing inhibitory circuits. Cell (2021).
7. Clarke, L.E. et al. Normal aging induces A1-like astrocyte reactivity. Proc Natl Acad Sci U S A 115, E1896-E1905 (2018).

Data exclusions

Exclusion criteria for experimental data points were sickness or death of animals during the experimental period. No outliers were excluded in this manuscript.

Replication

For each in vivo experimental condition, at least at least 3-5 animals were used per conditions. For in vitro experiments, at least two wells of cells or fields of view were included in each biological replicates. All attempts at replication were successful expect for the failures due to sickness and death of animals or technical faults during the experimental period.

Randomization

Mice and cells were randomized from each group.

Blinding

3D reconstruction was conducted blind.
Other data collection and analysis were not performed blind to the conditions of the experiments. For these experiments, mice were randomly assigned to control and experimental groups. Because morphological difference of microglia between control and PLX5622-administrated mice are apparently visible (at all-or-none level), no blinding was applied.

Reporting for specific materials, systems and methods

We require information from authors about some types of materials, experimental systems and methods used in many studies. Here, indicate whether each material, system or method listed is relevant to your study. If you are not sure if a list item applies to your research, read the appropriate section before selecting a response.

Materials & experimental systems

n/a	Involved in the study	
☐	☒	Antibodies
☒	☐	Eukaryotic cell lines
☒	☐	Palaeontology and archaeology
☐	☒	Animals and other organisms
☒	☐	Human research participants
☒	☐	Clinical data
☒	☐	Dual use research of concern

Methods

n/a	Involved in the study	
☒	☐	ChIP-seq
☐	☒	Flow cytometry
☒	☐	MRI-based neuroimaging

Antibodies

Antibodies used

Primary antibodies include goat anti-mCherry (Biorbyt, Cat#: Orb11618, Lot#: J2446; 1:500), rat anti-CD31 (BD Biosciences, Cat#: 550274, Lot#: 9259767; 1:10), rabbit anti-IBA1 (Wako, Cat#: 019-19741 Lot#: WDK2121; 1:500), goat anti-IBA1 (Abcam, Cat#: ab5076, Lot#: GR3187278-2; 1:500), chicken anti-GFP (Abcam, Cat#: ab13970, Lot#: GR236651-12; 1:1,000), rabbit anti-S100β (Abcam, Cat#: ab52642, Lot#: GR252937-5; 1:300), rabbit anti-GFAP (Abcam, Cat#: ab7260, Lot#: GR297722-2; 1:300) , rabbit anti-α-SMA (Abcam, Cat#: ab124964, Lot#: GR181740-66; 1:300), mouse anti-GFAP (Sigma-Aldrich, Cat#: G3893, Lot#: 105M4784V; 1:200), goat anti-PDGFR-β (R&D systems, Cat#: AF385, Lot#: BIWO619121; 1:200), rabbit anti-LC3 (Cell Signaling Technologies, Cat#: 4108, Lot#: 3; 1:100), rat anti-LAMP1 (1D4B) (Santa Cruz, Cat#: SC-19992, Lot#: C2715; 1:50), rabbit anti-PDGFR-α (Cell Signaling Technologies, Cat#: 3174s, Lot#: 7; 1:500), mouse anti-CC1 (Millipore, Cat#: 14-0661-82, Lot#: 3129980; 1:200), rat anti-mouse C4 (Abcam, Cat#: ab11863, Lot#: GR3315169-2; 1:100), goat anti-GFP (Abcam, Cat#: AB6673, Lot#: GR3373716-2; 1:1,000), chicken anti-mCherry (Abcam, Cat#: ab205402, Lot#: GR3271744-8; 1:500), chicken anti-NESTIN (Abcam, Cat#: ab134017, Lot#: GR3291127-1; 1:200), rabbit anti-RFP (Abcam, Cat#: ab62341, Lot#: GR3319727-1; 1:1,000), rabbit anti-NeuN (Abcam, Cat#: ab177487, Lot#: GR3275122-6; 1:500), rabbit anti-MBP (Abcam, Cat#: ab40390, Lot#: GR297609-1; 1:200), rabbit anti-LAMININ (Sigma, Cat#: L9393-100UL, Lot#: 087M4889V; 1:250), rabbit anti-GFP (Invitrogen, Cat#: A-11122, Lot#: 2273763; 1:1000), goat anti-OLIG2 (R&D, Cat#: AF2418, Lot#: UPA0719061; 1:400) and rabbit anti-PDGFα (Cell Signaling, Cat#: 3164S, Lot#: 02/2020-6; 1:500).

Secondary antibodies conjugated to Alexa Fluor 488 (AF488), AF568 and AF647 were 1:600 diluted if not specified, including AF488 donkey anti-rabbit (Thermo Fisher, Cat#: A11008, Lot#: 1829924), AF488 donkey anti-rabbit (Jackson ImmunoResearch, Cat#: 711-545-152, Lot#: 146247), AF488 donkey anti-goat (Jackson ImmunoResearch, Cat#: 705-545-003, Lot#: 145270, 1:2,000), AF488 donkey anti-goat (Thermo Fisher, Cat#: A11055, 1687906), AF488 donkey anti-chicken (Jackson ImmunoResearch, Cat#: 703-545-155, Lot#: 126602 and 122188), AF568 donkey anti-rabbit (Thermo Fisher, Cat#: A10042, Lot#: 1964370), AF568 donkey anti-goat (Thermo Fisher, Cat#: A11057, Lot#: 1871957 and 1640316), AF568 donkey anti-rat (Abcam, Cat#: Ab175475, Lot#: GR142910-1), AF488 goat anti-rat (Abcam, Cat#: Ab150157, Lot#: GR3189353-1) and AF647 donkey anti-rat (Jackson ImmunoResearch, Cat#: 712-605-153, Lot#: 142891).

4',6-diamidino-2-phenylindole (DAPI, 1:500, Sigma-Aldrich, Cat#: D9542-10MG, Lot#: 118M4025V) was utilized for visualizing the cell nucleus.

Validation

The validation data of each antibody are listed in the websites of corresponding manufactures. We validated the antibodies with positive controls and verified them based on the pattern and co-localization.

Animals and other organisms

Policy information about studies involving animals; ARRIVE guidelines recommended for reporting animal research

Laboratory animals

CX3CR1GFP/GFP (B6.129P-Cx3cr1tm1Litt/J, Stock No: 005582)37, CX3CR1-CreER (B6.129P2(C)-Cx3cr1tm2.1(cre/ERT2)Jung/J, Stock No: 020940)73, Ai14 (B6.Cg-Gt(ROSA)26Sortm14(CAG-tdTomato)Hze/J, Stock No: 007914)74, 5xFAD (B6.Cg-Tg (APPSwFlLon,PSEN1*M146L*L286V)6799Vas/Mmjax, Stock No: 34848-JAX)75, DTA (B6.129P2-Gt(ROSA)26Sortm1(DTA)Lky/J, aka Rosa26-DTA)76, C1qa-/- (B6(Cg)-C1qatm1d(EUCOMM)Wtsi/TennJ)51 and RUBICON-/- (C57BL/6-Rubcnem1Dgre/J)55 mice were purchased from The Jackson Laboratory. RUBICONfl/fl (B6/JGpt-Rubcnem1Cflox/Gpt) mice were purchased from GemPharmatech. NESTIN-GFP (Nes-GFP) mice were purchased from Cyagen Biosciences China41. C57BL/6J mice were purchased from Charles River (Beijing Vital River Laboratory Animal Technology). ALDH1L1-CreER mice were kindly donated from Prof. Tian-Ming Gao at Southern Medical University38.

C4b-/- mice were generated by CRISPR/Cas9-mediated genome editing. sgRNAs were designed using CHOPCHOP. Two selected sgRNAs targeting the exon 1 and 2 of all four C4b transcripts. GCTCCTCTGGGGGCTGGCCT and GCTGTACCCCCACCGACAGG were constructed by fusion of a T7 promoter and tracrRNA via overlapping PCR. Full length sgRNA was produced and transcribed. The transcribed sgRNA and Cas9 mRNA were both purified. The mixture of sgRNA (25 ng/μL each) and Cas9 mRNA (50 ng/μL) was microinjected into the cytoplasm of zygotes of C57BL/6 mice to generate C4b-/- mice. Fifth-five founder mice (F0) were born and analyzed for CRISPR-edited indels. Mouse tail clips were used for PCR amplification with primers flanking the sgRNAs region ACGCACATGCACAGGGACAC and TCAAGGCTGAGCAGCACAAA. The amplicons were directly Sanger sequenced (Supplemental Data 1), followed by CRISPR edit analysis of C4b indels using Synthego ICE CRISPR Analysis Tool (https://www.synthego.com/products/bioinformatics/crispr-analysis). For 55 F0 founder lines we generated, 8 were identified as C4b indel mutants with >83% of frameshift indels, including 5 biallelic lines (either homozygotes or compound heterozygotes) and 3 multiallelic mosaics.

P2Y12-CreER-GFP (P2ry12-p2A-CreER-p2A-EGFP) mice were generated by Beijing Biocytogen through the CRISPR/Cas9-based extreme genome editing (EGE) according to the authors' design, as a "fee-for-service". The p2A-CreERT2-p2A-EGFP cassette is

inserted between the last exon of P2ry12 gene (transcript 201), the gene encoding P2Y12, and its 3' UTR.

ALDH1L1-CreER mice were crossed with Ai14 mice to obtain ALDH1L1-CreER::Ai14 (ALDH1L1+/CreER::Ai14wt/mut) mice. ALDH1L1-CreER mice were crossed with Ai14 and RUBICONfl/fl to obtain ALDH1L1-CreER::Ai14::RUBICONfl/fl (ALDH1L1-CreER+/CreER::Ai14 wt/mut::RUBICONfl/fl) mice, aka RUBICON-cKO mice. CX3CR1-CreER mice were crossed with Ai14 mice to obtain CX3CR1-CreER::Ai14 (CX3CR1+/CreER::Ai14wt/mut) mice. P2Y12-CreER-GFP mice were crossed with Ai14 mice to obtain P2Y12-CreER-GFP::Ai14 (P2Y12+/CreER-GFP::Ai14wt/mut) mice. P2Y12-CreER-GFP mice were crossed with DTA mice to obtain P2Y12-CreER-GFP::DTA (P2Y12+/CreER-GFP::DTAwt/mut) mice. CX3CR1GFP/GFP mice were crossed with C57BL/6J to obtain CX3CR1+/GFP mice. P2Y12-CreER-GFP (P2Y12CreER-GFP/CreER-GFP) mice were crossed with C57BL/6J to obtain P2Y12+/CreER-GFP mice. Mice of both genders were utilized for experiments unless specified. All animals were housed in the specific pathogen-free (SPF) facility at Animal Facility of Shenzhen Institute of Advanced Technology at Chinese Academy of Sciences, Department of Laboratory Animal Science at Fudan University and Animal Facility of Shanghai Mental Health Center at Shanghai Jiao Tong University School of Medicine on a 12-and-12 h light-dark cycle with food and water ad libitum. The ambient temperature was kept at 20 °C to 26 °C and the humidity was maintained between 40% to 70%.

Wild animals	This study did not involve wild animals.
Field-collected samples	This study did not involve field-collected samples.
Ethics oversight	All mouse experiments were conducted in accordance with the guidelines of the Institutional Animal Care and Use Committee of Fudan University, Shenzhen Institute of Advanced Technology at Chinese Academy of Sciences and Shanghai Mental Health Center at Shanghai Jiao Tong University School of Medicine.

Note that full information on the approval of the study protocol must also be provided in the manuscript.

Flow Cytometry

Plots

Confirm that:

☒ The axis labels state the marker and fluorochrome used (e.g. CD4-FITC).

☒ The axis scales are clearly visible. Include numbers along axes only for bottom left plot of group (a 'group' is an analysis of identical markers).

☒ All plots are contour plots with outliers or pseudocolor plots.

☒ A numerical value for number of cells or percentage (with statistics) is provided.

Methodology

Sample preparation	For RNA-seq analysis of astrocyte, FACS was utilized to harvest tdTomato+ cells from tamoxifen-administered ALDH1L1-CreER::Ai14 mice. In brief, adult ALDH1L1-CreER::Ai14 mice were deep anaesthetized with a mixture of ketamine hydrochloride (100 mg per kg of body weight) and xylazine (10 mg per kg of body weight) by intraperitoneal injection. After that, animals were perfused with cold 1X PBS. Brains were then dissociated immediately and cut into 1 mm3 pieces by mouse stainless steel brain matrices (RWD). Next, tissue pieces were transferred into a C tube containing 3 mL papin (8 U/mL, Sangon Biotec, S501621) digestion buffer. The C tube was then attached onto a gentleMACS Octo Dissociator (Miltenyi) with 37C_ABDK program. At the end of the program, detached the C tube and briefly centrifuged at room temperature. Then, added 10 mL ice cold DBPS containing 0.5% BSA and pipetted up and down with 1 mL pipette until large tissue clumps were detached. Then filtered dissociated cells through a 70 μm cell strainer (Faclon). Centrifuged the cells at 300 g for 10 minutes at 4 ºC and discarded the supernatant. Resuspended cells in 4 mL 30% Percoll (Sigma-Aldrich) and centrifuged 700 g for 10 min at 4 ºC to remove debris. Washed the cell with 3 mL ice cold DBPS containing 0.5% BSA once and collected the dissociated cells for the subsequent cell sorting. Before loading the cells onto MoFlo Astrios EQ Cell Sorter (Beckman Coulter), cells were stained with pSIVA-FITC (Abcam) which was used for labeling death cells.
Instrument	MoFlo Astrios EQ Cell Sorter
Software	Cell sorter was controlled by Summit. FACS data were analyzed by FlowJo 10.4.
Cell population abundance	After removal the doublelets and cell debris by FSC/SSC, approximately 1.2 to 1.4 × 105 pSIVA-FITC- tdTomato+ cells were sorted for subsequent analysis.
Gating strategy	After removal the doublelets and cell debris by FSC/SSC, approximately 1.2 to 1.4 × 105 pSIVA-FITC- (488, 530/30) tdTomato+ (561, 585/42) cells were sorted for subsequent analysis with pSVIA-FITC.

☒ Tick this box to confirm that a figure exemplifying the gating strategy is provided in the Supplementary Information.
